# In Vitro and In Vivo Antimicrobial Activity of the Novel Peptide OMN6 against Multidrug-Resistant *Acinetobacter baumannii*

**DOI:** 10.3390/antibiotics11091201

**Published:** 2022-09-05

**Authors:** Janna Michaeli, Shira Mandel, Shelly Maximov, Jonathan Zazoun, Paola Savoia, Nimmi Kothari, Thomas Valmont, Livia Ferrari, Leonard R. Duncan, Stephen Hawser, Moshe Cohen-Kutner, Niv Bachnoff

**Affiliations:** 1 Omnix Medical Ltd., High-Tech Village, Givat-Ram Campus, Jerusalem 9270401, Israel; 2 Evotec Anti-Infective, Department of Microbiology Discovery, Aptuit (Verona) Srl, an Evotec Company, Via A. Fleming 4, 37135 Verona, Italy; 3 IHMA Europe Sàrl, Route de l’Ile-au-Bois 1A, 1870 Monthey, Switzerland; 4 JMI Laboratories, 345 Beaver Kreek Centre, Suite A, North Liberty, IA 52317, USA

**Keywords:** OMN6, antimicrobial peptides, antimicrobial resistance, AMR, *Acinetobacter baumannii*, infectious diseases

## Abstract

The rapid worldwide spread of antimicrobial resistance highlights the significant need for the development of innovative treatments to fight multidrug-resistant bacteria. This study describes the potent antimicrobial activity of the novel peptide OMN6 against a wide array of drug-resistant *Acinetobacter baumannii* clinical isolates. OMN6 prevented the growth of all tested isolates, regardless of any pre-existing resistance mechanisms. Moreover, in vitro serial-passaging studies demonstrated that no resistance developed against OMN6. Importantly, OMN6 was highly efficacious in treating animal models of lung and blood infections caused by multidrug-resistant *A. baumannii*. Taken together, these results point to OMN6 as a novel antimicrobial agent with the potential to treat life-threatening infections caused by multidrug-resistant *A. baumannii* avoiding resistance.

## 1. Introduction

Antimicrobial Resistance (AMR) has emerged as one of the leading public health threats in our times [1]. Crossing borders and pulling the world back to the pre-antibiotic era, AMR is endangering lives around the globe [2]. AMR is associated with a yearly death toll of 4.95 million people and if left unmitigated, may lead to the emergence of more lethal bacterial pathogens, thereby posing a staggering economic burden and costing significantly more lives [3].

Multi-Drug Resistant (MDR) bacteria such as *Acinetobacter baumannii* (AB) present an increasing sustained healthcare challenge. AB has been designated number one on the WHO Priority-Pathogen list since 2013 [4], with Carbapenem-Resistant AB (CRAB) already considered an ‘Urgent Threat’ by the CDC’s 2019 Antibiotic Resistance Threats Report [2]. As AMR spreads, we are left with an alarmingly small arsenal of effective treatments [5]. 

AB has the ability to acquire resistance through several mechanisms and this has led to the clinical emergence of strains that are resistant to almost all available antibiotics [6]. There are about 1.4 million AB infections per year globally, including an estimated ~200,000 infections per year in the developed western world (US/EU including Israel, Turkey, and Pacific OECD [Organisation for Economic Cooperation and Development] countries) [7]. CRAB accounts for 65% of AB-related pneumonia in the USA and Europe [8], and even after using appropriate empirical therapy, outcomes are very poor, resulting in ~50% mortality in our most modern ICUs [9].

Today, clinical approaches to treat MDR infections are generally limited to the repurposing of existing drugs and re-engineering small molecule-based antibiotics. This strategy is severely thwarted by the rise of AMR and the rapid emergence of resistant pathogens [10]. The available treatment options for AB are especially scarce and suffer from pharmacokinetic limitations such as high toxicity and low bioavailability. For example, the use of colistin is limited by renal toxicity [11]. Together with high rates of resistance development, it is clear that a new strategy is required to treat drug-resistant infections. New drugs, especially with novel mechanisms of action, are urgently needed to win the war against AMR [12,13,14].

OMN6 is a synthetic cyclic peptide composed of 40 amino acids that exerts a rapid bactericidal effect by causing the selective disruption of the bacterial membrane integrity [15]. OMN6 exhibits potent antimicrobial effects against susceptible and drug-resistant Gram-negative bacteria with no observable cross-resistance caused by common mechanisms of antimicrobial resistance [15]. Furthermore, OMN6 demonstrates enhanced resistance to proteolysis and no toxicity towards eukaryotic or mammalian cells [15].

In the current study, we present further in vitro and in vivo results that measure the antimicrobial activity of OMN6 against a wide range of AB clinical isolates. We demonstrate that OMN6 exhibits a low propensity for resistance development in vitro, that it has enhanced activity in the presence of lung-surfactant, and that it is efficacious in two murine models of severe bacterial infection caused by AB. The results support the continued development of OMN6 as a novel therapy to treat life-threatening infections caused by AB, such as bacteremia or severe pulmonary infections.

## 2. Results

The broad screening of OMN6 activity against 401 *A. baumannii* clinical isolates was tested. The objective of the study was to assess the activity of OMN6 against diverse AB clinical isolates with a wide spectrum of resistance patterns. Minimal Inhibitory Concentration (MIC) broth microdilution tests were performed by IHMA Europe (International Health Management Associates Europe; Monthey, Switzerland) on 401 AB clinical isolates that were collected worldwide from various infection types during the year 2019. As shown in Table 1, OMN6 was active against 100% of AB isolates with a very narrow MIC range (4–8 µg/mL) compared to the other antimicrobial agents tested. OMN6 activity was unaffected by the antimicrobial resistance mechanisms (including resistance to colistin) displayed by these AB isolates (see Supplementary Table S1), indicating that no resistance or cross-resistance against OMN6 was observed within the 401 AB clinical isolates.

**Lung surfactant effect on OMN6 in vitro activity.** In order to assess the potential impact of the lung environment on OMN6 activity, an MIC assay was conducted using 10 *A. baumannii-calcoaceticus* species complex clinical isolates by JMI Laboratories (North Liberty, IA, USA), in the presence of OMN6 alone, or OMN6 with 2.5% (*v*/*v*) Bovine Pulmonary Surfactant (BPS). Notably, the OMN6 MIC_50_ value against the AB isolate set decreased four-fold in 2.5% BPS (Table 2). This result demonstrates that the presence of BPS did not inhibit OMN6 and thus provides in vitro support for the use of OMN6 as a potential therapy for AB-based pulmonary infections.

**Figure 1:**
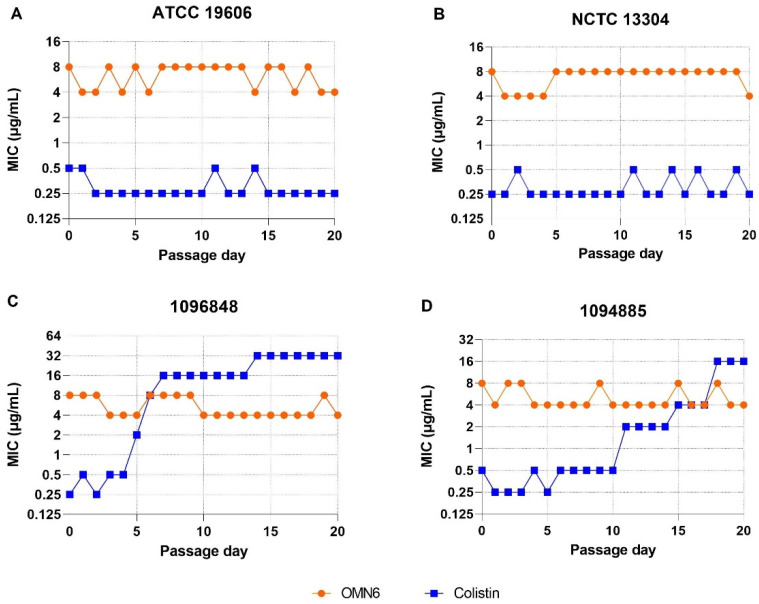
Serial passaging study with 4 *A. baumannii* QC strains and clinical isolates. The MIC assays were performed according to the broth microdilution method following the principles outlined in Clinical and Laboratory Standards Institute (CLSI) guidelines. During each serial passage, the contents from the well that contained the highest concentration of the compound producing visible growth during the previous day’s serial passage were sub-cultured and used to inoculate the next round of MIC panels, up to 20 serial passages. Two of the isolates tested were QC strains (ATCC 19606—panel (**A**); and NCTC 13304—panel (**B**)) and the two others were clinical isolates (1096848—panel (**C**) and 1094885—panel (**D**)). Supplementary information about the bacterial strains used in this study is displayed in Table S3.

**Serial passaging resistance studies with *A. baumannii* in the presence of OMN6.** To assess the potential for resistance development to OMN6 during the serial passaging of the *A. baumannii-calcoaceticus* species complex, two clinical isolates and two QC strains were incubated in the presence of sub-inhibitory concentrations of OMN6 for 20 passages. Colistin was used as a comparator drug for this study. Figure 1 demonstrates that the respective OMN6 MIC values remained unchanged (4–8 µg/mL) against the four strains tested over 20 days of passaging (panels A–D). By contrast, although the colistin MIC values against the two QC strains (panels A–B) remained stable during the entire experiment, the colistin MIC values against the two clinical isolates (panels C–D) increased dramatically from 0.25 to 16–32 µg/mL over the same time frame. Thus, we conclude that there was no evidence for the development of resistance by AB against the antimicrobial activity of OMN6.

**Figure 2:**
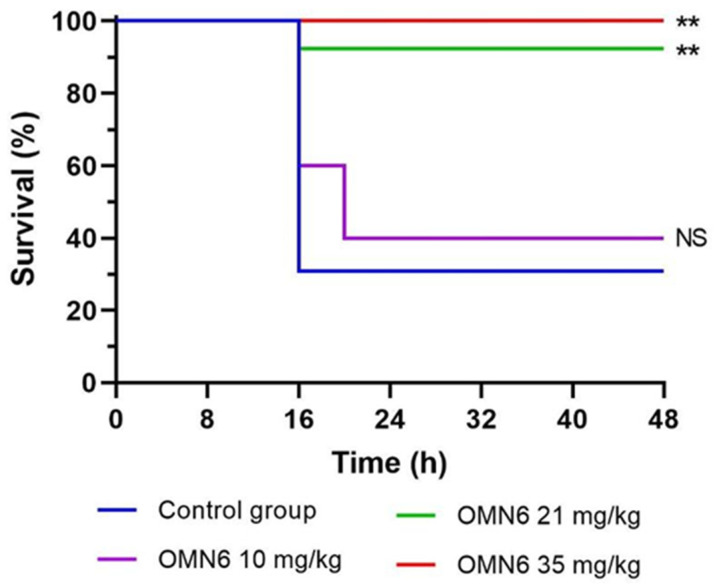
Efficacy of OMN6 in a mouse bacteremia survival model for up to 48 h. Neutropenic CD-1 female mice were intraperitoneally inoculated with *A. baumannii* MDR strain ATCC BAA-1793 (0.5 × 10^6^ CFU/mouse). Two hours post-inoculation, OMN6 was administered by an intravenous bolus of four separate doses (q1h) at 10, 21, or 35 mg/kg. OMN6 was administered only during the first 24 h of the experiment. Saline solution was administered for the control group (*n* ≥ 5, NS—Not Significant, ** *p* < 0.01; according to Log-rank (Mantel–Cox) statistical test. Supplementary information about the resistance pattern of the bacterial strain used in this study is displayed in Table S4.

**The efficacy of OMN6 intravenous treatment on survival in a lethal mouse bacteremia model using a multi-drug resistant *A. baumannii* strain.** A mouse bacteremia model was established to demonstrate the activity of OMN6 in a 48-h survival assay of mice with severe bloodstream infections. CD-1 female mice were intraperitoneally (IP) inoculated with 0.5 × 10^6^ cells of MDR AB strain ATCC BAA-1793. OMN6 was administered 2 h after bacterial inoculation as a split dose of four separate intravenous bolus injections (four doses × q1h) at 10, 21, or 35 mg/kg. The study results show that at 48 h post-inoculation, mice survival was 30% for the control group, whereas in the OMN6 treated groups of mice with 10 mg/kg, 21 mg/kg, and 35 mg/kg of OMN6, the survival fraction was 40%, 92%, and 100%, respectively (Figure 2). These results demonstrate that an intravenous administration of OMN6 protects mice from lethal bacteremia.

**Figure 3:**
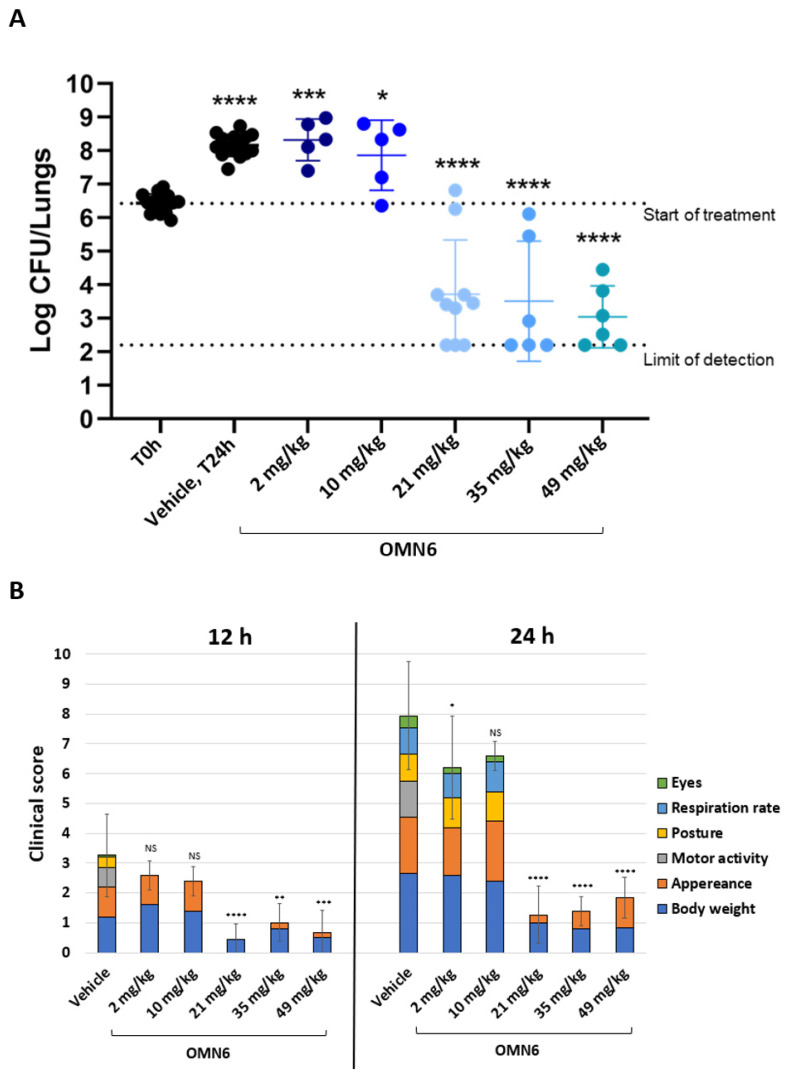
Bacterial burden in a mouse lung infection model induced by an MDR AB clinical isolate. (**A**) Lung bacterial burden. Neutropenic CD-1 male mice were intratracheally inoculated with MDR AB ACC000535 clinical isolate (1.5 × 10^6^ CFU/lungs). The treatment was administered by three intravenous bolus injections, one per hour (q1h), starting from 2 h post-infection (T0h), with doses of 2, 10, 21, 35, and 49 mg/kg. The control group (Veh) was administered with 0.9% normal saline solution. Bacterial burden in the lungs was evaluated 24 h post-infection. *n* = 15 untreated, *n* = 15 vehicle, *n* = 5, *n* = 5, *n* = 10, *n* = 6, and *n* = 6 OMN6-treated groups at 2, 10, 21, 35, and 49 mg/kg, respectively. Statistical analysis was performed by using one-way ANOVA followed by Dunnett’s post hoc test (*p* values vs. T0h, * *p* < 0.05, *** p < 0.001, **** *p* < 0.0001). (**B**) Clinical score. Six criteria (body weight, appearance, motor activity, posture, respiration rate, and eyes) to provide an overall score of 0 to 23 (*p* values vs. each group vehicle (12 h or 24 h): * *p* < 0.05, ** *p* < 0.01, *** *p* < 0.001, **** *p* < 0.0001; according to Student’s unpaired *t*-test). Supplementary information about the resistance pattern of the bacterial strains used in this study is displayed in Table S5.

**The efficacy of OMN6 intravenous treatment in the reduction in the bacterial burden in a mouse lung infection model using a multi-drug resistant *A. baumannii* strain.** A localized standard lung infection model (Evotec, Verona, Italy) was used in order to assess the reduction in the bacterial burden following a systemic (intravenous) administration of OMN6. For the induction of the lung infection, the MDR AB ACC000535 clinical isolate (resistant to imipenem, ciprofloxacin, cefotaxime, piperacillin/tazobactam, ceftazidime, gentamicin, amoxicillin/clavulanic acid) was used. Neutropenic CD-1 male mice were intratracheally (IT) inoculated with MDR AB ACC000535 bacterial suspension (1.5 × 10^6^ CFU/lungs) 2 h before the start of treatment. Then, OMN6 at doses of 2, 10, 21, 35, and 49 mg/kg was administered intravenously three times, once per hour (three doses × q1h). As shown in Figure 3A, 21 mg/kg OMN6 showed a bactericidal effect with a bacterial burden reduction greater than 2.7 log CFU/lungs after 24 h of treatment when compared to the untreated group (T0h), and a reduction of 4.4 log CFU/lungs when compared to the vehicle-treated group after 24 h. Importantly, the mean log CFU/lungs reduction increased with increasing OMN6 dose demonstrating a dose-dependent effect. Furthermore, the incidence of the clinical signs recorded was reduced in mice treated with 21 mg/kg OMN6 and above, compared to the vehicle-treated group (Figure 3B). Therefore, we conclude that 21 mg/kg OMN6 was the lowest tested efficacious dosing regimen in this animal model of MDR AB lung infection. 

**Figure 4:**
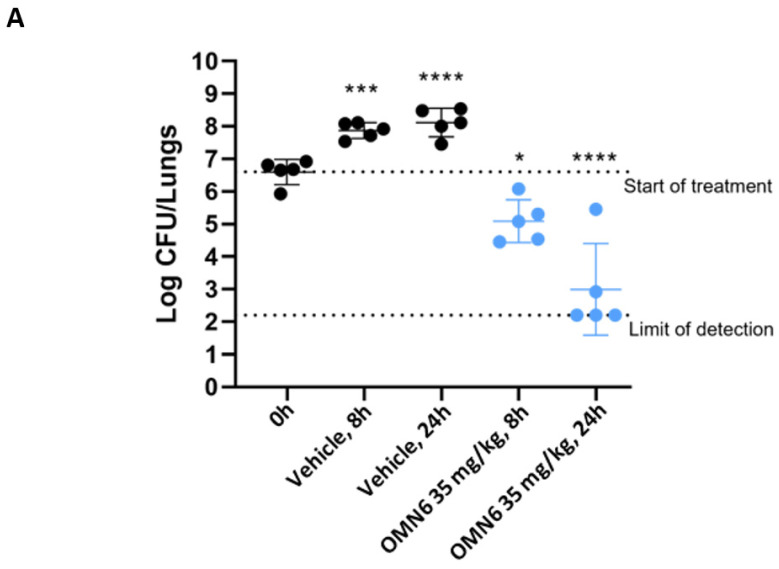

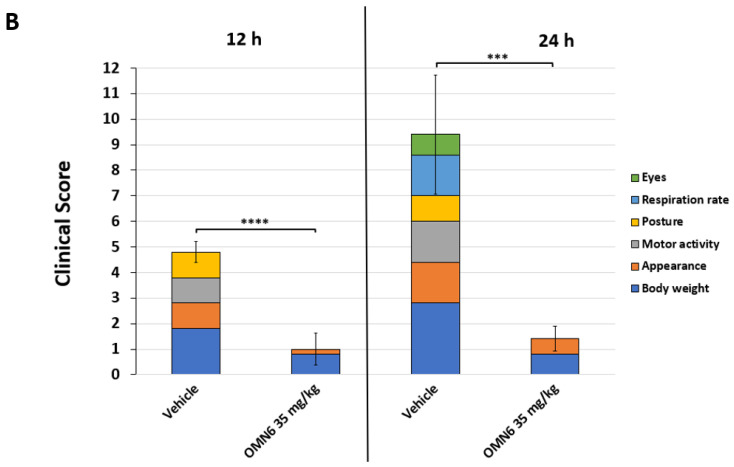
Time-course efficacy of OMN6 in a mouse lung infection model induced by an MDR AB clinical isolate. (**A**) Lung bacterial burden. Neutropenic CD-1 male mice were intratracheally inoculated with an MDR AB ACC000535 clinical isolate (2.7 × 10^6^ CFU/lungs). OMN6 at a dose of 35 mg/kg, or 0.9% Normal Saline solution (Vehicle), was administered by three intravenous bolus injections, one injection per hour (timepoints T0h, T1h, T2h), starting from 2 h post-infection (timepoint T0h). The bacterial burden was determined at 8 h (T8h) and 24 h (T24h) post-start of treatment. Statistical analysis was performed by using one-way ANOVA followed by Dunnett’s post hoc test (*n* = 5, *p* values vs. T0h, * *p* ˂ 0.05, *** *p* ˂ 0.001, **** *p* ˂ 0.0001). (**B**) Clinical score. Six criteria (body weight, appearance, motor activity, posture, respiration rate, and eyes) were assessed to provide an overall clinical score of 0 to 21 (*** *p* < 0.001, **** *p* < 0.0001; according to Student’s unpaired *t*-test). Supplementary information about the resistance pattern of the bacterial strains used in this study is displayed in Table S5.

The time course of OMN6 efficacy in a mouse lung infection model. An additional localized lung infection model was performed to determine the reduction in bacterial burden at two time points following an intravenous bolus systemic administration of OMN6, by Evotec, Verona, Italy. This experiment was performed in the same conditions as described in Figure 3, using the OMN6 dose of 35 mg/kg as full efficacy was observed with this dose both on the bacteremia and lung models, and thus allows to optimally explore the OMN6 killing kinetics. OMN6 was intravenously administered three times, once per hour (three doses × q1h), starting from 2 h post-infection (T0h) with MDR AB ACC000535 clinical isolate. As shown in Figure 4A, OMN6 exhibited rapid bactericidal activity against the AB strain: by 8 h post-start of treatment, the initial bacteria burden had decreased by 1.51 log CFU/lungs, and by 24 h, the initial bacteria burden had decreased by 3.08 log CFU/lungs versus the start of treatment. Furthermore, animals treated with OMN6 showed less severe clinical signs after 12 h and 24 h of treatment compared to the vehicle-treated group at each time point (Figure 4B). Since there was no additional administration of OMN6 after 2 h from the first one, the results demonstrate that the OMN6 bactericidal effect continues for at least 22 h after the last drug administration, reflecting a clinical improvement in the treated animals compared to the vehicle group. 

## 3. Discussion

The results presented in this study highlight the disruptive features of the novel engineered peptide OMN6. Developed as an innovative therapy against life-threatening bacterial infections caused by MDR *A. baumannii*, OMN6 employs a membrane disruption mode-of-action that avoids resistance [15].

When tested against a large set of contemporary AB clinical isolates, OMN6 in vitro antimicrobial activity was unaffected by various pre-existing resistance mechanisms, including carbapenem resistance and colistin resistance. Among the comparator antimicrobials tested, only colistin displayed significant in vitro activity against this isolate set. Colistin, also called polymyxin E, is an old antibiotic of renewed interest as a last-resort therapy due to its high in vitro activity against carbapenem-resistant, Gram-negative bacteria [16,17]. However, the use of colistin as standard-of-care treatment is limited by its nephrotoxic and neurotoxic effects [11], and by existing and spreading resistance [18,19,20], which can be due to chromosomal mutations or the acquisition of mobile genetic elements containing resistance genes like *mcr-1*. Indeed, we observed the rapid development of colistin resistance, likely due to chromosomal mutations, during serial passaging experiments with AB clinical isolates.

In contrast, no resistance developed against OMN6 during in vitro serial passaging experiments, not in colistin-susceptible nor in colistin-resistant strains. These observations confirm the low propensity for the development of resistance against OMN6, which is most likely due to its non-conventional membrane-disrupting mode of action [15].

In further microbiological studies aimed at understanding OMN6 activity in the lungs, we observed that OMN6 antimicrobial activity was enhanced in the presence of Bovine Pulmonary Surfactant (BPS). This effect may be due to the synergistic effect of OMN6 with surfactant proteins presenting antimicrobial properties [21,22]. These results provide a key advantage to OMN6 over many currently available antibiotics that lose their antimicrobial efficiency in the presence of lung surfactant [23,24,25,26].

As for every drug candidate, the in vitro to in vivo translation is a crucial step to confirm if an agent is active in the context of a whole organism. In the case of OMN6 design, special focus was put on the following parameters: (1) the stability of OMN6 to overcome the in vivo proteolysis that barred other antimicrobial peptides from being active in in vivo systems [27]; (2) the availability of OMN6 and its ability to reach the site of infection following IV dosing; and (3) the retention of OMN6 activity in a mammalian organism despite the presence of proteases, lung surfactants, and the organisms’ natural clearance mechanisms.

All these parameters have been confirmed by the efficacy of OMN6 in two different MDR AB infection models in mice: a bacteremia/survival model and a lung infection model. In these two models, it was established that OMN6 was efficacious in vivo because a single-day intravenous therapy regimen with OMN6 was enough to prevent mortality, to reduce the bacterial burden in the lungs, and to improve dramatically the clinical symptoms of the infected animals. These effects can be directly attributed to OMN6, as increased OMN6 doses showed greater antimicrobial effects in the animals.

Additionally, the bactericidal activity in the lung model increased between 8 and 24 h, which suggests a long-lasting antimicrobial effect. This activity pattern reduces the probability for resistance development and for a bacterial population rebound. These observations suggest that future OMN6 treatment in the clinics may allow clinicians to achieve efficacy while avoiding resistance. We are currently extending this work by investigating the PK/PD properties and safety profile of OMN6 in multiple in vitro and in vivo systems. A better understanding of these parameters will be beneficial to define adequately the best clinical administration parameters.

## 4. Methods

OMN6 peptide. OMN6 was synthesized by Ambiopharm Inc. (North Augusta, SC, USA) for Omnix Medical Ltd. (Jerusalem, Israel). The therapeutic doses used in the in vivo studies were adjusted to the real OMN6 content in the lyophilized powder as calculated with the following formula:

OMN6 content (%)=Net Peptide Content (%)∗Purity (%)

where the Net Peptide Content is the fraction of the peptidic material (OMN6 and peptidic impurities) quantified by elemental analysis (%N), and Purity is the area of the OMN6 main peak as obtained by reverse-phase high-performance liquid chromatography (RP-HPLC).

Broad Minimal Inhibitory Concentration (MIC) screening of OMN6 activity against clinical isolates of *Acinetobacter baumannii*. The study was carried out by IHMA Europe Sàrl (Monthey, Switzerland), a subsidiary of International Health Management Associates Inc. (Schaumburg, IL, USA). MIC tests were performed by broth microdilution in Difco Mueller-Hinton broth (MHB; product code #275730; Becton Dickinson, Franklin Lakes, NJ, USA) following the principles outlined in the Clinical and Laboratory Standards Institute (CLSI) guidelines [28]. Bacterial species were identified at IHMA Europe using standard microbiology methods and matrix-assisted laser desorption ionization-time of flight mass spectrometry (MALDI-TOF MS; Bruker Daltonics, Bremen, Germany). Bacterial inocula were prepared at a concentration of 1 × 10^6^ CFU/mL by 100-fold dilution from a 0.5 McFarland suspension in MHB. Antimicrobial panels, containing 50 μL antimicrobial solutions at two-fold their final concentrations, were diluted with 50 μL of inoculum to give a final cell density of 5 × 10^5^ CFU/mL and the desired test concentrations of antimicrobial agents. The test plates were incubated, and the MIC value of each compound was determined as the lowest concentration that completely inhibited the growth of the organism in the microdilution well, as detected by the unaided eye. A total of 401 *A. baumannii* clinical isolates were tested. In addition, *A. baumannii* Bouvet and Grimont ATCC 19606 (American Type Culture Collection; Manassas, VA, USA) and NCTC 13304 (National Collection of Type Cultures; Colindale, UK) were tested as quality control (QC) strains. MIC_50_ and MIC_90_ represent the lowest MIC values required to inhibit the growth of 50% and 90% of the isolates tested, respectively. MIN and MAX represent the minimum and maximum MIC values determined for each antibiotic tested with the clinical isolates, respectively. The resistance pattern for each antibiotic is presented according to breakpoint values as determined by CLSI [29]. Supplementary information about the resistance pattern of each clinical isolate used in this study is presented in Table S1, and supplementary data about the MIC distributions for all antibiotics are presented in Table S2.

Lung surfactant effect on OMN6 in vitro activity. The study was performed by JMI Laboratories (North Liberty, IA, USA) to determine the effect of Bovine Pulmonary Surfactant (BPS) on the in vitro activity of OMN6 against *A. baumannii* clinical isolates. A total of 10 *A. baumannii-calcoaceticus* species complex isolates were recovered from documented infections during the year 2019. The isolates were identified by the submitting laboratories and confirmed by JMI Laboratories using standard microbiology methods and matrix-assisted laser desorption ionization-time of flight mass spectrometry (Bruker Daltonics, Bremen, Germany). The isolates were tested for antimicrobial susceptibility using broth microdilution methodology according to CLSI guidelines. The testing medium was Difco Mueller–Hinton broth (MHB; product code #275730; Beckton Dickinson, Franklin Lakes, NJ, USA). For QC purposes, cation-adjusted Mueller–Hinton broth (CA-MHB; Becton Dickinson, Franklin Lakes, NJ, USA) was used to test susceptibility for colistin and meropenem. The isolates were tested with OMN6 alone or in the presence of 2.5% (*v*/*v*) BPS (Survanta^®^; AbbVie, Lake Bluff, IL, USA). Supplementary information about the resistance pattern of the bacterial strains used in this study is displayed in Table S3. As a control, we demonstrated that the MIC value for daptomycin against *Staphylococcus aureus* ATCC 29213 was greatly increased in the presence of 2.5% (*v*/*v*) BPS (data not shown; as a reference, see [25]).

Serial-passaging resistance studies with OMN6. The study was performed at JMI Laboratories (North Liberty, IA, USA) to assess the in vitro development of resistance to OMN6 and colistin during the serial passaging of *A. baumannii* strains and clinical isolates. Single-step in vitro resistance-selection studies could not be conducted with OMN6 because OMN6 activity was abrogated on solid growth media. Bacterial species were identified by JMI Laboratories using standard microbiology methods and matrix-assisted laser desorption ionization-time of flight mass spectrometry (MALDI-TOF MS; Bruker Daltonics, Bremen, Germany). Strains and clinical isolates were tested for antimicrobial susceptibility using broth microdilution methodology according to breakpoint values as determined by CLSI [29]. The passaging medium used for the tests was Difco Mueller–Hinton broth (MHB); for quality control purposes, cation-adjusted Mueller–Hinton broth (CA-MHB; Becton Dickinson, Franklin Lakes, NJ, USA) was used to test susceptibility to colistin. Susceptibility was determined according to the European Committee on Antimicrobial Susceptibility Testing (EUCAST) breakpoint values for colistin against *A. baumannii* isolates [30]. On day 1 of serial passaging, each strain and isolate were tested against OMN6 and colistin in MIC panels produced using MHB broth and an initial starting inoculum of approximately 5 × 10^5^ CFU/mL. A serial passage is defined as the inoculation of bacterial contents from the sub-inhibitory concentration, which is the highest concentration of compound at which bacterial growth was observed during the previous day. The inoculation was performed by transferring approximately 100 μL of culture from the selected well into fresh MHB, followed by incubation at 35 °C for 1.5–3 h until the culture reached the turbidity of a 0.5 McFarland standard. The culture was diluted and used to inoculate the next round of MIC panels. This process was repeated for 20 daily transfers, and the apparent MIC values were tracked daily. The terminal phenotypes were stable after passaging on drug-free agar and re-testing. Supplementary information about the bacterial strains used in this study is displayed in Table S3.

Efficacy of OMN6 intravenous treatment on survival in a lethal mouse bacteremia model using an MDR AB strain. The survival study was performed at Omnix Medical with neutropenic CD-1 female mice (Envigo, Indianapolis, IN, USA), weighing 21–25 g. The bacterial inoculum was prepared by suspending a few colonies of MDR AB ATCC BAA-1793 (OMN6 MIC = 8 µg/mL) in 5 mL of Difco Nutrient Broth (Beckton Dickinson, Franklin Lakes, NJ, USA), which was incubated with shaking for 2 h at 37 °C. After the incubation, the inoculum was adjusted to 0.5 McFarland turbidity standard. The bacterial suspension was diluted in Difco Nutrient Broth and the mice were intraperitoneally (IP) inoculated with 100 µL bacterial suspension at a concentration of 0.5 × 10^6^ CFU/mouse with 7.5% (*w*/*v*) mucin. Infection was confirmed 1 h after inoculum by testing blood bacterial burden using tail blood. OMN6 was administrated 2 h post-infection as a split dose of four separate doses as intravenous bolus injections at 10, 21, or 35 mg/kg, one injection per hour (four doses × q1h at timepoints 2 h, 3 h, 4 h, 5 h from inoculation). The control group was administrated with saline solution at the same time points as the treated mice. Briefly, 48 h after infection, the mice were sacrificed according to endpoints established by the Authority for Biological and Biomedical Models (AAALAC accredited program) by IP injection of 100 µL Ketamine–Xylazine (from a 0.9% saline solution of 1 mL with 10 mg Ketamine and 5 mg Xylazine). The number of animals per group was as follows: *n* = 13 for the control group, *n* = 5, *n* = 13, and *n* = 7 for treated mice with 10, 21, and 35 mg/kg OMN6, respectively. Statistical analysis was performed according to the Log-rank (Mantel–Cox) test. Supplementary information about the bacterial strain used in this study is displayed in Table S4.

Efficacy of OMN6 intravenous treatment in the reduction in the bacterial burden in a mouse lung infection model induced by MDR AB. The study was performed by Evotec (Verona, Italy). Male CD-1 mice (Charles River, Italy), weighing between 18–20 g at arrival, were acclimatized for a period of 5 days before any experimental procedure. Animals were monitored during the entire period of the studies and clinical signs were recorded. Briefly, 62 animals were rendered neutropenic by two intraperitoneal injections of cyclophosphamide (Sigma-Aldrich, Italy) at 150 and 100 mg/kg, four and one day before the infection, respectively. On the day of the infection, the bacterial challenge was prepared by suspending a few colonies of MDR AB ACC00535 (clinical isolate from Evotec EvostrAIn culture collection; (OMN6 MIC = 4 µg/mL)) in a sterile physiological 0.9% saline solution to reach the turbidity of a 0.5 McFarland. The mice were anesthetized using inhaled 2.5% isoflurane and intratracheally inoculated with 50 μL of the bacterial suspension corresponding to 1.5 × 10^6^ CFU/lungs. Two hours post-infection, treatment was administrated by three intravenous bolus injections of 2, 10, 21, 35, or 49 mg/kg OMN6, one injection per hour (three doses × q1h). The animals were sacrificed by overdose of inhaled 5% isoflurane two hours post-infection (untreated group, start of treatment) and at the end of the study (all treated groups 24 h post-start of treatment). The lungs were collected, weighed, and homogenized with Cryolys^®^ Evolution Precellys system (Bertin Instruments, Montigny–le–Bretonneux, France). Dilutions of lung homogenate were plated on tryptic-soy agar plates and incubated overnight for lung bacterial burden determination. The mice were monitored during the entire study and scored for signs of infection at 12 and 24 h post-infection, according to the criteria presented in Table 3. 

The survival rate was measured at 24 h post-infection. Statistical analysis was performed according to One-way ANOVA followed by Dunnett’s post hoc test. Supplementary information about the bacterial strain used in this study is displayed in Table S5.

Time course of OMN6 efficacy in a mouse lung infection model. The study was performed by Evotec (Verona, Italy) with neutropenic CD–1 male mice (Charles River, Italy), weighing 18–20 g at arrival (see the previous section). On the day of infection, the bacterial challenge was prepared as previously reported. Twenty-five mice were anesthetized and intratracheally inoculated with 50 µL of the bacterial suspension corresponding to 2.7 × 10^6^ CFU/lungs. Treatment started two hours post-infection (T = 0) and the animals were injected with three intravenous bolus injections with saline (control group, *n* = 10) or 35 mg/kg OMN6 (*n* = 10), one injection per hour (three doses × q1h). the Animals (*n* = 5/group/timepoint) were sacrificed by overdose of inhaled 5% isoflurane at 0 (untreated), 8, and 24 h post-start of treatment. The lungs were collected and homogenized with Cryolys^®^ Evolution Precellys system (Bertin Instruments, Montigny-le-Bretonneux, France). Dilutions of lung homogenates were plated on Tryptic-Soy agar plates and incubated overnight for bacterial burden determination. During the study, the mice were scored for signs of infection at 12 and 24 h post-start of treatment, according to the clinical scores presented above. Statistical analysis was performed according to One–way ANOVA followed by Dunnett’s post hoc test.

## 5. Conclusions

Taken together, the data presented in this article support the continued development of OMN6 as a novel therapy for severe infections caused by MDR *A. baumannii*, regardless of pre-existing resistance. Blood-Stream Infections (BSI) and Hospital-Acquired or Ventilator-Associated Bacterial Pneumonia (HABP/VABP) are areas of specific interest due to the limited therapeutic options available and the rise in AMR. Pending clinical tolerability, pharmacokinetics, and efficacy in clinical trials, OMN6 may be well positioned to become a first-line therapy for severe and life-threatening infections caused by *A. baumannii*.

## Figures and Tables

**Table 1:** Minimal Inhibitory Concentration (MIC) results against 401 *A. baumannii* isolates for OMN6 and comparator antibiotics. The bacteria were subjected to MIC tests by broth microdilution method following the principles outlined in Clinical and Laboratory Standards Institute (CLSI) guidelines. MIC**_50_** and MIC**_90_** represent the lowest MIC value required to inhibit the growth of 50% and 90% of the isolates tested, respectively. MIN and MAX represent the minimum and maximum MIC values determined for each antibiotic tested with the clinical isolates, respectively. The resistance phenotype, determined by CLSI breakpoints and EUCAST interpretive criteria, is summarized as a percentage in the last column according to the following abbreviations: S—Sensitive, I—Intermediate, R—Resistant, SAM—Ampicillin/Sulbactam (2:1), FEP—Cefepime, CAZ—Ceftazidime, CRO—Ceftriaxone, COL—Colistin, GEN—Gentamicin, LVX—Levofloxacin, MEM—Meropenem, TET—Tetracycline, SXT—Trimethoprim/Sulfamethoxazole (1:19), and N/A—not available. Supplementary information about the resistance phenotype of each clinical isolate used in this study is displayed in Table S1, and supplementary data about the MIC distribution for all antibiotics are presented in Table S2.

	MIC (µg/mL)					Percentage		
Drug	MIC_50_	MIC_90_	MIN	MAX	CLSI Breakpoints (S|I|R)	S	I	R
**OMN6**	4	8	4	8	N/A	-	-	-
**SAM (2:1)**	16	64	≤1	>64	≤8/4│16/8│≥32/16	39.2	15.7	45.1
**FEP**	32	>64	≤0.25	>64	≤8│16│≥32	32.9	12.5	54.6
**CAZ**	64	>64	≤0.25	>64	≤8│16│≥32	27.7	4.0	68.3
**CRO**	>64	>64	1	>64	≤8│16–32│≥64	15.2	12.2	72.6
**COL**	0.25	0.5	≤0.12	>8	≤2│-│≥4	97.3	-	2.7
**GEN**	>16	>16	≤0.12	>16	≤4│8│≥16	41.9	1.2	56.9
**LVX**	4	16	≤0.12	>32	≤2│4│≥8	44.4	16.0	39.7
**MEM**	>16	>16	≤0.06	>16	≤2│4│≥8	29.9	1.5	68.6
**TET**	8	>32	≤0.12	>32	≤4│8│≥16	47.1	3.2	49.6
**SXT (1:19)**	4	>32	≤0.06	>32	≤2/38│-│≥4/76	46.9	-	53.1

**Table 2:** Modal MIC values of OMN6 against 10 *A. baumannii* clinical isolates with or without addition of 2.5% (*v*/*v*) Bovine Pulmonary Surfactant (BPS). Bacteria were subjected to MIC tests by broth microdilution method following the principles outlined in Clinical and Laboratory Standards Institute (CLSI) guidelines. The mode of ≥3 MIC values for each strain/testing condition combination is shown. Supplementary information about the resistance pattern of the bacterial strains used in this study is displayed in Table S3.

Treatment	No. and Cumulative % of *A. baumannii* Isolates Inhibited at MIC (mg/L) of:						
	1	2	4	8	16	MIC_50_	MIC_90_
**OMN6**			0 0.0	9 90.0	1 100.0	8	8
**OMN6 with 2.5% (*v*/*v*) BPS**	0 0.0	7 70.0	1 80.0	1 90.0	1 100.0	2	8

**Table 3:** Scoring system for mice clinical signs’ assessment.

Variable	Score and Description
Body Weight	0—Weight loss < 5%
	1—Weight loss 5–10%
	2—Weight loss 11–15%
	3—Weight loss 16–20%
	4—Weight loss > 20%
Appearance	0—Smooth coat
	1—Patches of hair piloerected
	2—Majority of back piloerected
	3—Piloerection, mouse appears “puffy”
	4—Piloerection, mouse appears emaciated
	5—Skin lesions, such as lump, ulcer
Activity	0—Normal activity
	1—Slightly reduced activity
	2—Marked reduced activity
	3—Severely impaired activity
Posture	0—Normal
	1—Slightly hunched, moving freely
	2—Hunched with activity
	3—Hunched without activity
	4—Ventral/lateral decubitus
Respiration Rate	0—Normal, rapid mouse respiration
	1—Slightly decreased respiration
	2—Moderately reduced respiration
	3—Severely reduced respiration
	4—Asphyxia
Eyes	0—Opened
	1—Eyes not fully opened, possibly with secretions
	2—Eyes half closed or more, possibly with secretions
	3—Eyes closed or milky

## Data Availability

The data presented in this study may be available on request from the corresponding author. The data are not publicly available due to privacy and third-party restrictions.

## Supplementary Materials

The following are available online at https://www.mdpi.com/article/10.3390/antibiotics11091201/s1, Table S1: Supplementary information about the resistance pattern of 401 *A. baumannii* clinical isolates used in the MIC study presented in Table 1; Table S2: MIC distribution against 401 clinical isolates tested for OMN6 and other antibiotics; Table S3: Supplementary information about the *A. baumannii-calcoaceticus* species complex strains and clinical isolates tested in the serial passaging study presented in Figure 1 and the MIC study presented in Table 2; Table S4: Supplementary information about the resistance pattern of the bacterial strain *A. baumannii* BAA-1793 used in the mouse bacteremia survival model presented in Figure 2; Table S5: Supplementary information about the resistance pattern of the *A. baumannii* ACC000535 clinical isolate used in the chronic lung infection model presented in Figure 3 and in the time-course study presented in Figure 4.
