# Supplementary material for: In Vitro and In Vivo Antimicrobial Activity of the Novel Peptide OMN6 against Multidrug-Resistant Acinetobacter baumannii
Source: Antibiotics (Basel). 2022 Sep 5;11(9):1201. doi: 10.3390/antibiotics11091201 (PMC9494975; doi:10.3390/antibiotics11091201)
Supplement: Supplementary file 1 [file antibiotics-11-01201-s001.zip › antibiotics-1895408-supplementary.pdf]

**TITLE**

# In Vitro and In Vivo Antimicrobial Activity of the Novel Peptide OMN6 against Multidrug-Resistant *Acinetobacter baumannii*

**AUTHORS**

**Janna Michaeli [1], Shira Mandel [1], Shelly Maximov [1], Jonathan Zazoun [1], Paola Savoia [2], Nimmi Kothari [3], Thomas Valmont [3], Livia Ferrari [2], Leonard R. Duncan [4], Stephen Hawser [3] and Moshe Cohen-Kutner [1] and Niv Bachnoff [1,*]**

**AFFILIATIONS**

1 Omnix Medical Ltd., High-Tech Village, Givat-Ram Campus, 9270401 Jerusalem, Israel

2 Evotec Anti-Infective, Department of Microbiology Discovery, Aptuit (Verona) Srl, an Evotec Company, via A. Fleming 4, 37135 Verona, Italy

3 IHMA Europe Sàrl, Route de l'Ile-au-Bois 1A, 1870 Monthey, Switzerland

4 JMI Laboratories, 345 Beaver Kreek Centre, Suite A, North Liberty, IA 52317, USA

* Correspondence: niv@omnixmedical.com

**Table S1. Supplementary information about the resistance pattern of 401 *A. baumannii* clinical isolates used in the Minimal Inhibitory Concentration (MIC) study presented in Table 1.** The resistance phenotype of the different clinical isolates was characterized by broth microdilution method by IHMA Europe Sàrl (Monthey, Switzerland). The study followed the principles and breakpoint values outlined in Clinical and Laboratory Standards Institute (CLSI) and European Committee on Antimicrobial Susceptibility Testing (EUCAST) guidelines. **Green: susceptible isolates**. **Yellow/Orange: intermediate isolates. Red: resistant isolates.** The resistance pattern is presented according to the following abbreviations: *S* Susceptible, *I* Intermediate, *R* Resistant, *SAM* Ampicillin/Sulbactam (2:1), *FEP* Cefepime, *CAZ* Ceftazidime, *CRO* Ceftriaxone, *COL* Colistin, *GEN* Gentamicin, *LVX* Levofloxacin, *MEM* Meropenem, *TET* Tetracycline, *SXT* Trimethoprim/Sulfamethoxazole (1:19), *N/A* Not available.

| | | | | | MIC values (µg/mL) | | | | | | | | | | |
|---|---|---|---|---|---|---|---|---|---|---|---|---|---|---|---|
| | | | | **CLSI Cut-off Values (µg/mL)** | N/A | S≤4/8 R≥16/32 | S≤8 R≥32 | S≤8 R≥32 | S≤8 R≥64 | S≤2 R≥8 | S≤4 R≥16 | S≤2 R≥8 | S≤2 R≥8 | S≤4 R≥16 | S≤2/38 R≥4/76 |
| **IHMA Clinical Isolate #** | **Organism** | **Year Collected** | **Country** | **Body Location** | **OMN6** | **SAM (2:1)** | **FEP** | **CAZ** | **CRO** | **COL** | **GEN** | **LVX** | **MEM** | **TET** | **TSX (1:19)** |
| 2040459 | *A. baumannii* | 2019 | Kenya | Respiratory: Endotracheal aspirate | 8 | 2 | 2 | 4 | 16 | 0.25 | 0.5 | ≤0.12 | 0.12 | 0.5 | ≤0.06 |
| 2040348 | *A. baumannii* | 2019 | Kenya | Respiratory: Endotracheal aspirate | 8 | 4 | 2 | 4 | 16 | 0.5 | 0.5 | ≤0.12 | 0.12 | 0.5 | 0.12 |
| 1997827 | *A. baumannii* | 2019 | Morocco | Genitourinary: Urine | 4 | 16 | 32 | 64 | >64 | 0.25 | >16 | 2 | >16 | 4 | 2 |
| 2040416 | *A. baumannii* | 2019 | Kenya | Respiratory: Endotracheal aspirate | 4 | 8 | 8 | >64 | >64 | 0.25 | >16 | 2 | 0.25 | >32 | 4 |
| 2039312 | *A. baumannii* | 2019 | Kenya | Gastrointestinal: Other | 4 | 16 | 32 | >64 | >64 | 0.25 | >16 | 4 | 16 | >32 | 0.5 |
| 2039379 | *A. baumannii* | 2019 | Kenya | Respiratory: Other | 8 | ≤1 | ≤0.25 | 1 | 2 | 0.25 | 0.5 | ≤0.12 | ≤0.06 | 0.25 | 0.25 |
| 2015128 | *A. baumannii* | 2019 | South Africa | Bodily Fluids: Peritoneal | 4 | 8 | 2 | 4 | 16 | 0.25 | >16 | 1 | 8 | 0.5 | 8 |

| | | | | | MIC values (μg/mL) | | | | | | | | | | | |
|---|---|---|---|---|---|---|---|---|---|---|---|---|---|---|---|---|
| | | | | CLSI Cut-off Values (μg/mL) | N/A | S≤4/8 R≥16/32 | S≤8 R≥32 | S≤8 R≥32 | S≤8 R≥64 | S≤2 R≥8 | S≤4 R≥16 | S≤2 R≥8 | S≤2 R≥8 | S≤4 R≥16 | S≤2/38 R≥4/76 |
| **IHMA Clinical Isolate #** | **Organism** | **Year Collected** | **Country** | **Body Location** | **OMN6** | **SAM (2:1)** | **FEP** | **CAZ** | **CRO** | **COL** | **GEN** | **LVX** | **MEM** | **TET** | **TSX (1:19)** |
| 2039416 | *A. baumannii* | 2019 | Kenya | Respiratory: Other | 4 | 16 | 32 | 64 | >64 | 0.25 | >16 | 16 | >16 | >32 | >32 |
| 2040243 | *A. baumannii* | 2019 | Kenya | Genitourinary: Urine | 8 | 32 | >64 | >64 | >64 | 0.25 | >16 | ≤0.12 | 1 | 1 | >32 |
| 1997739 | *A. baumannii* | 2019 | Morocco | Cardiovascular: Blood | 4 | ≤1 | 1 | 1 | 8 | ≤0.12 | 0.25 | ≤0.12 | 0.25 | 0.5 | 0.12 |
| 2014931 | *A. baumannii* | 2019 | South Africa | Respiratory: Sputum | 4 | >64 | 1 | 2 | 8 | ≤0.12 | 0.5 | ≤0.12 | ≤0.06 | 0.5 | 0.12 |
| 2039368 | *A. baumannii* | 2019 | Kenya | Respiratory: Sputum | 4 | 16 | 32 | >64 | >64 | 0.5 | >16 | 4 | 16 | >32 | 0.5 |
| 2007187 | *A. baumannii* | 2019 | Morocco | Respiratory: Bronchoalveolar lavage | 4 | >64 | >64 | >64 | >64 | ≤0.12 | 0.25 | 4 | >16 | 1 | 4 |
| 2040386 | *A. baumannii* | 2019 | Kenya | Respiratory: Endotracheal aspirate | 8 | 2 | 2 | 4 | 16 | 0.5 | 0.5 | ≤0.12 | 0.25 | 0.5 | 0.12 |
| 2015016 | *A. baumannii* | 2019 | South Africa | Respiratory: Endotracheal aspirate | 4 | 16 | 16 | 4 | 16 | 0.25 | 0.5 | ≤0.12 | 16 | 0.5 | 0.25 |
| 1997921 | *A. baumannii* | 2019 | Morocco | Respiratory: Other | 4 | 2 | 2 | 4 | 16 | 0.25 | 0.25 | ≤0.12 | ≤0.06 | 0.5 | 0.12 |
| 2040420 | *A. baumannii* | 2019 | Kenya | Cardiovascular: Blood | 4 | 4 | 32 | >64 | >64 | 0.25 | >16 | 2 | 2 | >32 | >32 |
| 2032922 | *A. baumannii* | 2019 | South Africa | Respiratory: Endotracheal aspirate | 4 | ≤1 | 0.5 | 1 | 2 | ≤0.12 | 0.5 | ≤0.12 | ≤0.06 | 0.5 | ≤0.06 |
| 2032966 | *A. baumannii* | 2019 | South Africa | Respiratory: Sputum | 4 | >64 | >64 | >64 | >64 | 0.25 | >16 | 2 | >16 | >32 | >32 |

| | | | | MIC values (µg/mL) | | | | | | | | | | | |
|---|---|---|---|---|---|---|---|---|---|---|---|---|---|---|---|
| | | | | **CLSI Cut-off Values (µg/mL)** | N/A | S≤4/8 R≥16/32 | S≤8 R≥32 | S≤8 R≥32 | S≤8 R≥64 | S≤2 R≥8 | S≤4 R≥16 | S≤2 R≥8 | S≤2 R≥8 | S≤4 R≥16 | S≤2/38 R≥4/76 |
| **IHMA Clinical Isolate #** | **Organism** | **Year Collected** | **Country** | **Body Location** | **OMN6** | **SAM (2:1)** | **FEP** | **CAZ** | **CRO** | **COL** | **GEN** | **LVX** | **MEM** | **TET** | **TSX (1:19)** |
| 2039270 | *A. baumannii* | 2019 | Kenya | Gastrointestinal: Other | 4 | >64 | >64 | >64 | >64 | 0.25 | 0.25 | ≤0.12 | >16 | >32 | 0.12 |
| 1966965 | *A. baumannii* | 2019 | South Africa | Respiratory: Endotracheal aspirate | 8 | 16 | 64 | >64 | >64 | >8 | >16 | 32 | >16 | >32 | >32 |
| 2040278 | *A. baumannii* | 2019 | Kenya | Genitourinary: Urine | 4 | 16 | 32 | 64 | >64 | ≤0.12 | 0.25 | 2 | >16 | >32 | 2 |
| 2032963 | *A. baumannii* | 2019 | South Africa | Cardiovascular: Blood | 4 | ≤1 | 1 | 2 | 8 | ≤0.12 | ≤0.12 | ≤0.12 | 0.12 | 0.5 | 0.12 |
| 2034657 | *A. baumannii* | 2019 | Tunisia | Respiratory: Endotracheal aspirate | 4 | 2 | 1 | 4 | 16 | 0.25 | 0.5 | ≤0.12 | 0.25 | 0.5 | 0.12 |
| 2039197 | *A. baumannii* | 2019 | Kenya | Cardiovascular: Blood | 8 | 32 | 64 | >64 | >64 | 1 | >16 | 4 | >16 | >32 | 16 |
| 2032577 | *A. baumannii* | 2019 | South Africa | Cardiovascular: Blood | 4 | >64 | >64 | >64 | >64 | 0.25 | >16 | 2 | >16 | >32 | >32 |
| 2013283 | *A. baumannii* | 2019 | Tunisia | Respiratory: Endotracheal aspirate | 8 | 64 | >64 | >64 | >64 | 0.25 | >16 | 8 | >16 | >32 | 1 |
| 1997743 | *A. baumannii* | 2019 | Morocco | Cardiovascular: Blood | 4 | 2 | 2 | 4 | 32 | 0.25 | 0.5 | ≤0.12 | ≤0.06 | 0.5 | ≤0.06 |
| 2032730 | *A. baumannii* | 2019 | South Africa | Respiratory: Sputum | 8 | 32 | 32 | >64 | >64 | 0.5 | >16 | 4 | >16 | >32 | 8 |
| 1997983 | *A. baumannii* | 2019 | Morocco | Respiratory: Other | 4 | 16 | 32 | >64 | >64 | 0.25 | >16 | 8 | >16 | 4 | 16 |
| 2013248 | *A. baumannii* | 2019 | Tunisia | Respiratory: Endotracheal aspirate | 4 | 32 | 32 | 32 | >64 | 0.25 | >16 | 8 | >16 | 8 | >32 |

| | | | | | MIC values (µg/mL) | | | | | | | | | | | |
|---|---|---|---|---|---|---|---|---|---|---|---|---|---|---|---|---|
| | | | | CLSI Cut-off Values (µg/mL) | N/A | S≤4/8 R≥16/32 | S≤8 R≥32 | S≤8 R≥32 | S≤8 R≥64 | S≤2 R≥8 | S≤4 R≥16 | S≤2 R≥8 | S≤2 R≥8 | S≤4 R≥16 | S≤2/38 R≥4/76 |
| **IHMA Clinical Isolate #** | **Organism** | **Year Collected** | **Country** | **Body Location** | **OMN6** | **SAM (2:1)** | **FEP** | **CAZ** | **CRO** | **COL** | **GEN** | **LVX** | **MEM** | **TET** | **TSX (1:19)** |
| 2007234 | *A. baumannii* | 2019 | Morocco | Respiratory: Bronchoalveolar lavage | 8 | 16 | 32 | 64 | >64 | 0.25 | 0.25 | 1 | 16 | 32 | 0.25 |
| 1997910 | *A. baumannii* | 2019 | Morocco | Respiratory: Other | 4 | >64 | >64 | >64 | >64 | 0.25 | >16 | 4 | >16 | 2 | 0.12 |
| 2007306 | *A. baumannii* | 2019 | Morocco | Respiratory: Bronchoalveolar lavage | 4 | 32 | 32 | 64 | >64 | 0.25 | 16 | 4 | 16 | >32 | >32 |
| 2007119 | *A. baumannii* | 2019 | Morocco | Genitourinary: Urine | 4 | 32 | 32 | 32 | >64 | 0.25 | >16 | 4 | 16 | 4 | 32 |
| 2007176 | *A. baumannii* | 2019 | Morocco | Respiratory: Bronchoalveolar lavage | 8 | 64 | 32 | >64 | >64 | 8 | >16 | 16 | 16 | >32 | 32 |
| 2014712 | *A. baumannii* | 2019 | South Africa | Genitourinary: Urine | 4 | 8 | 8 | 4 | 16 | ≤0.12 | 16 | 4 | 16 | >32 | 8 |
| 2007305 | *A. baumannii* | 2019 | Morocco | Respiratory: Bronchoalveolar lavage | 4 | 32 | 32 | 64 | >64 | 0.25 | >16 | 2 | >16 | >32 | 32 |
| 2007304 | *A. baumannii* | 2019 | Morocco | Respiratory: Bronchoalveolar lavage | 4 | 16 | 32 | 64 | >64 | 0.25 | 2 | 8 | >16 | >32 | >32 |
| 2007242 | *A. baumannii* | 2019 | Morocco | Respiratory: Bronchoalveolar lavage | 4 | 8 | 16 | >64 | >64 | 0.25 | 4 | 4 | 16 | 4 | 0.25 |
| 2015096 | *A. baumannii* | 2019 | South Africa | Bodily Fluids: Peritoneal | 4 | 8 | 8 | 2 | 16 | ≤0.12 | 0.5 | ≤0.12 | 8 | 0.25 | 0.12 |
| 2032588 | *A. baumannii* | 2019 | South Africa | Cardiovascular: Blood | 8 | 2 | 1 | 4 | 16 | 0.25 | 0.5 | ≤0.12 | 0.12 | 0.5 | 0.12 |

| | | | | MIC values (µg/mL) | | | | | | | | | | | |
|---|---|---|---|---|---|---|---|---|---|---|---|---|---|---|---|
| | | | | **CLSI Cut-off Values (µg/mL)** | N/A | S≤4/8 R≥16/32 | S≤8 R≥32 | S≤8 R≥32 | S≤8 R≥64 | S≤2 R≥8 | S≤4 R≥16 | S≤2 R≥8 | S≤2 R≥8 | S≤4 R≥16 | S≤2/38 R≥4/76 |
| **IHMA Clinical Isolate #** | **Organism** | **Year Collected** | **Country** | **Body Location** | **OMN6** | **SAM (2:1)** | **FEP** | **CAZ** | **CRO** | **COL** | **GEN** | **LVX** | **MEM** | **TET** | **TSX (1:19)** |
| 2013258 | *A. baumannii* | 2019 | Tunisia | Respiratory: Endotracheal aspirate | 4 | 32 | 32 | 32 | >64 | 0.25 | >16 | 8 | >16 | 8 | >32 |
| 2034667 | *A. baumannii* | 2019 | Tunisia | Respiratory: Endotracheal aspirate | 4 | 32 | 64 | >64 | >64 | ≤0.12 | 0.5 | 2 | >16 | >32 | ≤0.06 |
| 2034594 | *A. baumannii* | 2019 | Tunisia | Respiratory: Endotracheal aspirate | 4 | 16 | 64 | >64 | >64 | 0.25 | >16 | >32 | 16 | >32 | 1 |
| 2013240 | *A. baumannii* | 2019 | Tunisia | Respiratory: Endotracheal aspirate | 4 | 8 | 32 | >64 | >64 | 0.25 | 0.5 | 1 | 16 | 0.5 | ≤0.06 |
| 2032673 | *A. baumannii* | 2019 | South Africa | Respiratory: Endotracheal aspirate | 4 | 16 | 16 | 4 | 16 | 0.25 | 1 | ≤0.12 | 16 | 1 | 0.12 |
| 2100758 | *A. baumannii* | 2019 | Japan | Genitourinary: Urine | 4 | ≤1 | 0.5 | 1 | 2 | 0.25 | 0.5 | ≤0.12 | ≤0.06 | 0.25 | 0.12 |
| 1966920 | *A. baumannii* | 2019 | South Africa | Genitourinary: Urine | 4 | 16 | 32 | 64 | >64 | 0.25 | 2 | 4 | >16 | >32 | >32 |
| 2100740 | *A. baumannii* | 2019 | Japan | Genitourinary: Urine | 4 | ≤1 | 0.5 | 1 | 4 | 0.25 | 0.5 | ≤0.12 | 0.12 | 0.25 | 0.12 |
| 2034598 | *A. baumannii* | 2019 | Tunisia | Respiratory: Endotracheal aspirate | 4 | >64 | >64 | >64 | >64 | ≤0.12 | >16 | 2 | >16 | 2 | 8 |
| 2038542 | *A. baumannii* | 2019 | Korea, South | Respiratory: Sputum | 4 | 16 | 32 | 64 | >64 | 0.25 | >16 | 2 | >16 | >32 | 32 |
| 2032729 | *A. baumannii* | 2019 | South Africa | Respiratory: Endotracheal aspirate | 4 | 2 | 2 | 4 | 16 | 0.25 | 0.5 | ≤0.12 | 0.12 | 0.5 | 0.12 |

| | | | | MIC values (µg/mL) | | | | | | | | | | | |
|---|---|---|---|---|---|---|---|---|---|---|---|---|---|---|---|
| | | | | **CLSI Cut-off Values (µg/mL)** | N/A | S≤4/8 R≥16/32 | S≤8 R≥32 | S≤8 R≥32 | S≤8 R≥64 | S≤2 R≥8 | S≤4 R≥16 | S≤2 R≥8 | S≤2 R≥8 | S≤4 R≥16 | S≤2/38 R≥4/76 |
| **IHMA Clinical Isolate #** | **Organism** | **Year Collected** | **Country** | **Body Location** | **OMN6** | **SAM (2:1)** | **FEP** | **CAZ** | **CRO** | **COL** | **GEN** | **LVX** | **MEM** | **TET** | **TSX (1:19)** |
| 2013284 | *A. baumannii* | 2019 | Tunisia | Respiratory: Endotracheal aspirate | 4 | 16 | 64 | 32 | >64 | 0.25 | 16 | 2 | >16 | 4 | 8 |
| 2034561 | *A. baumannii* | 2019 | Tunisia | Cardiovascular: Blood | 4 | >64 | >64 | >64 | >64 | 0.25 | >16 | 4 | >16 | >32 | 0.5 |
| 1990182 | *A. baumannii* | 2019 | Korea, South | Respiratory: Bronchoalveolar lavage | 8 | 16 | 32 | >64 | >64 | 0.25 | >16 | 8 | >16 | 4 | 16 |
| 1966954 | *A. baumannii* | 2019 | South Africa | Respiratory: Sputum | 4 | >64 | >64 | >64 | >64 | >8 | >16 | 2 | >16 | 16 | >32 |
| 2013290 | *A. baumannii* | 2019 | Tunisia | Respiratory: Endotracheal aspirate | 8 | 64 | 64 | >64 | >64 | 0.25 | >16 | 16 | >16 | >32 | 2 |
| 2034602 | *A. baumannii* | 2019 | Tunisia | Respiratory: Endotracheal aspirate | 4 | 8 | 16 | 32 | >64 | 0.25 | >16 | 16 | 16 | >32 | 32 |
| 2034368 | *A. baumannii* | 2019 | Taiwan | Gastrointestinal: Gall Bladder | 4 | ≤1 | 1 | 2 | 8 | 0.25 | 0.5 | ≤0.12 | 0.12 | 0.25 | 0.12 |
| 2015116 | *A. baumannii* | 2019 | South Africa | Bodily Fluids: Peritoneal | 4 | 16 | 16 | 4 | 16 | 0.25 | 0.5 | ≤0.12 | 16 | 0.5 | 0.12 |
| 1995938 | *A. baumannii* | 2019 | Japan | Gastrointestinal: Gall Bladder | 4 | ≤1 | 1 | 1 | 2 | ≤0.12 | 0.25 | ≤0.12 | ≤0.06 | 0.25 | ≤0.06 |
| 2032693 | *A. baumannii* | 2019 | South Africa | Respiratory: Endotracheal aspirate | 8 | 16 | 32 | 32 | >64 | 0.25 | 2 | 4 | 16 | >32 | >32 |
| 2033023 | *A. baumannii* | 2019 | South Africa | Respiratory: Sputum | 4 | 32 | 64 | >64 | >64 | ≤0.12 | >16 | 16 | >16 | >32 | >32 |
| 2074737 | *A. baumannii* | 2019 | Malaysia | Bodily Fluids: Peritoneal | 4 | >64 | 16 | >64 | >64 | 0.25 | >16 | 2 | 1 | >32 | 0.5 |

| | | | | | MIC values (µg/mL) | | | | | | | | | | |
|---|---|---|---|---|---|---|---|---|---|---|---|---|---|---|---|
| | | | | **CLSI Cut-off Values (µg/mL)** | N/A | S≤4/8 R≥16/32 | S≤8 R≥32 | S≤8 R≥32 | S≤8 R≥64 | S≤2 R≥8 | S≤4 R≥16 | S≤2 R≥8 | S≤2 R≥8 | S≤4 R≥16 | S≤2/38 R≥4/76 |
| **IHMA Clinical Isolate #** | **Organism** | **Year Collected** | **Country** | **Body Location** | **OMN6** | **SAM (2:1)** | **FEP** | **CAZ** | **CRO** | **COL** | **GEN** | **LVX** | **MEM** | **TET** | **TSX (1:19)** |
| 2058689 | *A. baumannii* | 2019 | Taiwan | Respiratory: Sputum | 8 | ≤1 | 1 | 2 | 16 | 0.25 | 0.5 | ≤0.12 | ≤0.06 | 0.25 | ≤0.06 |
| 2013202 | *A. baumannii* | 2019 | Tunisia | Cardiovascular: Blood | 4 | 32 | 32 | >64 | >64 | 0.25 | >16 | 4 | >16 | 8 | >32 |
| 2136953 | *A. baumannii* | 2019 | Thailand | Gastrointestinal: Gall Bladder | 4 | 16 | 16 | 64 | >64 | 0.25 | >16 | 2 | 16 | 2 | 8 |
| 2034597 | *A. baumannii* | 2019 | Tunisia | Respiratory: Endotracheal aspirate | 4 | 32 | 64 | >64 | >64 | 0.25 | >16 | 2 | >16 | >32 | 0.5 |
| 1985098 | *A. baumannii* | 2019 | Thailand | Respiratory: Sputum | 4 | 64 | 64 | >64 | >64 | 0.25 | >16 | 2 | >16 | >32 | 32 |
| 2046641 | *A. baumannii* | 2019 | Taiwan | Respiratory: Endotracheal aspirate | 4 | 2 | 1 | 2 | 8 | ≤0.12 | 0.5 | ≤0.12 | 0.12 | 0.5 | 0.12 |
| 1993482 | *A. baumannii* | 2019 | Thailand | Gastrointestinal: Gall Bladder | 4 | 64 | 64 | >64 | >64 | 0.25 | >16 | 8 | >16 | >32 | >32 |
| 1985099 | *A. baumannii* | 2019 | Thailand | Respiratory: Sputum | 4 | >64 | >64 | >64 | >64 | 0.25 | >16 | 2 | >16 | >32 | 32 |
| 2008218 | *A. baumannii* | 2019 | Belgium | Cardiovascular: Blood | 4 | ≤1 | 1 | 2 | 8 | 0.25 | 0.25 | ≤0.12 | 0.12 | 0.5 | 0.12 |
| 1987077 | *A. baumannii* | 2019 | Croatia | Cardiovascular: Blood | 8 | 16 | 16 | >64 | >64 | 0.25 | 2 | 2 | >16 | >32 | 32 |
| 1970291 | *A. baumannii* | 2019 | Korea, South | Genitourinary: Urine | 8 | 64 | 64 | 64 | >64 | 0.25 | >16 | 8 | >16 | 2 | 32 |
| 2020550 | *A. baumannii* | 2019 | Croatia | Respiratory: Endotracheal aspirate | 4 | 16 | 16 | 64 | >64 | 0.25 | >16 | 2 | >16 | >32 | >32 |
| 2058692 | *A. baumannii* | 2019 | Taiwan | Respiratory: Sputum | 4 | 64 | 64 | 16 | >64 | 0.25 | >16 | 32 | 16 | >32 | >32 |

| | | | | CLSI Cut-off Values (µg/mL) | N/A | S≤4/8 R≥16/32 | S≤8 R≥32 | S≤8 R≥32 | S≤8 R≥64 | S≤2 R≥8 | S≤4 R≥16 | S≤2 R≥8 | S≤2 R≥8 | S≤4 R≥16 | S≤2/38 R≥4/76 |
|---|---|---|---|---|---|---|---|---|---|---|---|---|---|---|---|
| | | | | | MIC values (µg/mL) | | | | | | | | | | |
| **IHMA Clinical Isolate #** | **Organism** | **Year Collected** | **Country** | **Body Location** | **OMN6** | **SAM (2:1)** | **FEP** | **CAZ** | **CRO** | **COL** | **GEN** | **LVX** | **MEM** | **TET** | **TSX (1:19)** |
| 2038537 | *A. baumannii* | 2019 | Korea, South | Respiratory: Bronchoalveolar lavage | 4 | 8 | 16 | 64 | >64 | 0.25 | >16 | 16 | 16 | 2 | 8 |
| 1987181 | *A. baumannii* | 2019 | Croatia | Respiratory: Endotracheal aspirate | 8 | 64 | 64 | >64 | >64 | 1 | >16 | 2 | >16 | >32 | 1 |
| 2007738 | *A. baumannii* | 2019 | Thailand | Gastrointestinal: Gall Bladder | 4 | >64 | >64 | >64 | >64 | 0.25 | >16 | 16 | >16 | >32 | >32 |
| 1987202 | *A. baumannii* | 2019 | Croatia | Respiratory: Endotracheal aspirate | 4 | 16 | 32 | >64 | >64 | 0.25 | >16 | 8 | >16 | >32 | 2 |
| 1984211 | *A. baumannii* | 2019 | Korea, South | Respiratory: Sputum | 4 | 32 | 64 | >64 | >64 | 0.25 | >16 | 8 | >16 | 4 | 16 |
| 1990247 | *A. baumannii* | 2019 | Korea, South | Respiratory: Sputum | 4 | 32 | 32 | >64 | >64 | ≤0.12 | 1 | 2 | 16 | 2 | 32 |
| 1970277 | *A. baumannii* | 2019 | Korea, South | Genitourinary: Urine | 8 | 32 | 64 | 64 | >64 | 0.25 | >16 | 8 | >16 | 4 | 16 |
| 2046756 | *A. baumannii* | 2019 | Taiwan | Gastrointestinal: Gall Bladder | 4 | 8 | 8 | 64 | >64 | 0.25 | >16 | 32 | 8 | >32 | 4 |
| 2037902 | *A. baumannii* | 2019 | Thailand | Respiratory: Sputum | 4 | 8 | 16 | 32 | >64 | 0.25 | >16 | 2 | 8 | >32 | 16 |
| 1993544 | *A. baumannii* | 2019 | Thailand | Genitourinary: Urine | 4 | 8 | 32 | >64 | >64 | ≤0.12 | 0.25 | 2 | 8 | 0.5 | ≤0.06 |
| 1985097 | *A. baumannii* | 2019 | Thailand | Respiratory: Endotracheal aspirate | 4 | ≤1 | 1 | 2 | 16 | ≤0.12 | 0.25 | ≤0.12 | 0.12 | 1 | 0.12 |
| 1999178 | *A. baumannii* | 2019 | Belgium | Cardiovascular: Blood | 4 | ≤1 | 1 | 2 | 8 | 0.25 | 0.5 | ≤0.12 | 0.12 | 0.25 | ≤0.06 |

| | | | | | | MIC values (µg/mL) | | | | | | | | | |
|---|---|---|---|---|---|---|---|---|---|---|---|---|---|---|---|
| | | | | **CLSI Cut-off Values (µg/mL)** | N/A | S≤4/8 R≥16/32 | S≤8 R≥32 | S≤8 R≥32 | S≤8 R≥64 | S≤2 R≥8 | S≤4 R≥16 | S≤2 R≥8 | S≤2 R≥8 | S≤4 R≥16 | S≤2/38 R≥4/76 |
| **IHMA Clinical Isolate #** | **Organism** | **Year Collected** | **Country** | **Body Location** | **OMN6** | **SAM (2:1)** | **FEP** | **CAZ** | **CRO** | **COL** | **GEN** | **LVX** | **MEM** | **TET** | **TSX (1:19)** |
| 1993545 | *A. baumannii* | 2019 | Thailand | Genitourinary: Urine | 4 | 64 | >64 | >64 | >64 | ≤0.12 | >16 | 4 | 16 | >32 | >32 |
| 2008178 | *A. baumannii* | 2019 | Belgium | Genitourinary: Urine | 4 | >64 | >64 | >64 | >64 | 0.25 | >16 | 8 | >16 | >32 | 32 |
| 1957635 | *A. baumannii* | 2019 | Thailand | Respiratory: Sputum | 4 | 16 | 32 | >64 | >64 | ≤0.12 | 0.25 | 2 | 16 | >32 | ≤0.06 |
| 1995202 | *A. baumannii* | 2019 | Czech Republic | Respiratory: Sputum | 4 | ≤1 | 0.5 | 1 | 2 | 0.25 | 0.5 | ≤0.12 | ≤0.06 | 0.25 | 0.12 |
| 1987190 | *A. baumannii* | 2019 | Croatia | Respiratory: Endotracheal aspirate | 4 | 64 | 64 | >64 | >64 | 0.5 | >16 | 4 | >16 | >32 | 1 |
| 2020393 | *A. baumannii* | 2019 | Croatia | Respiratory: Other | 8 | 32 | 64 | >64 | >64 | 0.25 | >16 | 8 | >16 | >32 | >32 |
| 2007739 | *A. baumannii* | 2019 | Thailand | Gastrointestinal: Gall Bladder | 4 | 2 | 1 | 2 | 8 | 0.25 | 0.5 | ≤0.12 | 8 | 0.5 | 0.12 |
| 2020344 | *A. baumannii* | 2019 | Croatia | Respiratory: Endotracheal aspirate | 4 | 64 | 64 | >64 | >64 | 0.25 | >16 | 2 | >16 | >32 | 8 |
| 1957634 | *A. baumannii* | 2019 | Thailand | Respiratory: Sputum | 4 | 16 | 64 | >64 | >64 | ≤0.12 | 0.5 | 1 | 16 | >32 | ≤0.06 |
| 1995229 | *A. baumannii* | 2019 | Czech Republic | Respiratory: Sputum | 4 | 32 | 64 | >64 | >64 | 0.25 | >16 | 8 | >16 | >32 | >32 |
| 1957646 | *A. baumannii* | 2019 | Thailand | Genitourinary: Urine | 4 | 16 | 16 | 64 | >64 | 0.5 | >16 | 2 | >16 | >32 | 16 |
| 1987171 | *A. baumannii* | 2019 | Croatia | Respiratory: Sputum | 4 | 32 | 64 | >64 | >64 | 1 | >16 | 2 | >16 | >32 | 1 |
| 2031463 | *A. baumannii* | 2019 | Greece | Cardiovascular: Blood | 4 | 64 | >64 | >64 | >64 | 2 | >16 | 4 | >16 | >32 | 32 |

| | | | | | | MIC values (μg/mL) | | | | | | | | | |
|---|---|---|---|---|---|---|---|---|---|---|---|---|---|---|---|
| | | | | CLSI Cut-off Values (μg/mL) | N/A | S≤4/8 R≥16/32 | S≤8 R≥32 | S≤8 R≥32 | S≤8 R≥64 | S≤2 R≥8 | S≤4 R≥16 | S≤2 R≥8 | S≤2 R≥8 | S≤4 R≥16 | S≤2/38 R≥4/76 |
| **IHMA Clinical Isolate #** | **Organism** | **Year Collected** | **Country** | **Body Location** | **OMN6** | **SAM (2:1)** | **FEP** | **CAZ** | **CRO** | **COL** | **GEN** | **LVX** | **MEM** | **TET** | **TSX (1:19)** |
| 1957655 | *A. baumannii* | 2019 | Thailand | Genitourinary: Urine | 4 | 32 | >64 | >64 | >64 | ≤0.12 | 0.5 | 2 | 16 | 16 | ≤0.06 |
| 2065587 | *A. baumannii* | 2019 | France | Genitourinary: Urine | 4 | ≤1 | 1 | 2 | 8 | 0.25 | 0.25 | ≤0.12 | 0.12 | 0.5 | 0.12 |
| 1989553 | *A. baumannii* | 2019 | Germany | Respiratory: Endotracheal aspirate | 4 | ≤1 | 0.5 | 0.5 | 4 | 0.25 | 0.5 | ≤0.12 | 0.12 | 0.5 | 0.25 |
| 1989607 | *A. baumannii* | 2019 | Germany | Respiratory: Endotracheal aspirate | 4 | ≤1 | 0.5 | 2 | 8 | 0.25 | 0.5 | ≤0.12 | ≤0.06 | 0.25 | ≤0.06 |
| 1995161 | *A. baumannii* | 2019 | Czech Republic | Genitourinary: Kidneys | 4 | ≤1 | 1 | 2 | 8 | 0.25 | 0.5 | ≤0.12 | 0.12 | 0.5 | 0.25 |
| 2065297 | *A. baumannii* | 2019 | Germany | Respiratory: Bronchial brushing | 4 | 4 | 8 | 16 | >64 | ≤0.12 | 2 | 8 | 0.5 | >32 | 1 |
| 1983239 | *A. baumannii* | 2019 | Greece | Respiratory: Endotracheal aspirate | 8 | 32 | 64 | >64 | >64 | >8 | >16 | 4 | >16 | 2 | 32 |
| 1974367 | *A. baumannii* | 2019 | Germany | Genitourinary: Urinary Bladder | 4 | 2 | 2 | 4 | 16 | 0.25 | 0.5 | ≤0.12 | 0.12 | 0.5 | 0.12 |
| 2039091 | *A. baumannii* | 2019 | Hungary | Cardiovascular: Blood | 8 | 64 | 16 | >64 | >64 | 0.25 | >16 | 16 | 8 | 4 | 32 |
| 1993543 | *A. baumannii* | 2019 | Thailand | Genitourinary: Urine | 4 | 64 | 64 | >64 | >64 | 0.25 | >16 | 2 | >16 | >32 | 0.5 |
| 2038976 | *A. baumannii* | 2019 | Hungary | Bodily Fluids: Peritoneal | 4 | 32 | 32 | >64 | >64 | 0.25 | >16 | 8 | >16 | >32 | >32 |
| 2078200 | *A. baumannii* | 2019 | Italy | Cardiovascular: Blood | 4 | 32 | 32 | 64 | >64 | 0.25 | >16 | 32 | >16 | >32 | >32 |

| | | | | MIC values (µg/mL) | | | | | | | | | | | |
|---|---|---|---|---|---|---|---|---|---|---|---|---|---|---|---|
| | | | | **CLSI Cut-off Values (µg/mL)** | N/A | S≤4/8 R≥16/32 | S≤8 R≥32 | S≤8 R≥32 | S≤8 R≥64 | S≤2 R≥8 | S≤4 R≥16 | S≤2 R≥8 | S≤2 R≥8 | S≤4 R≥16 | S≤2/38 R≥4/76 |
| **IHMA Clinical Isolate #** | **Organism** | **Year Collected** | **Country** | **Body Location** | **OMN6** | **SAM (2:1)** | **FEP** | **CAZ** | **CRO** | **COL** | **GEN** | **LVX** | **MEM** | **TET** | **TSX (1:19)** |
| 1987198 | *A. baumannii* | 2019 | Croatia | Respiratory: Endotracheal aspirate | 8 | 32 | 64 | 64 | >64 | 0.5 | >16 | 4 | >16 | >32 | 32 |
| 2118994 | *A. baumannii* | 2019 | France | Gastrointestinal: Other | 4 | ≤1 | 1 | 2 | 8 | ≤0.12 | 0.5 | ≤0.12 | 0.12 | 0.25 | ≤0.06 |
| 1987189 | *A. baumannii* | 2019 | Croatia | Respiratory: Endotracheal aspirate | 8 | 32 | 64 | >64 | >64 | 0.5 | >16 | 4 | >16 | >32 | 1 |
| 2020464 | *A. baumannii* | 2019 | Croatia | Cardiovascular: Blood | 4 | 32 | 16 | >64 | >64 | 0.25 | >16 | 2 | >16 | 4 | 2 |
| 2020542 | *A. baumannii* | 2019 | Croatia | Respiratory: Endotracheal aspirate | 8 | 8 | 4 | >64 | >64 | ≤0.12 | >16 | 1 | >16 | 0.5 | 0.5 |
| 2065104 | *A. baumannii* | 2019 | Germany | Gastrointestinal: Other | 4 | 8 | 16 | 4 | 16 | 0.25 | >16 | 2 | 16 | 32 | 8 |
| 2020348 | *A. baumannii* | 2019 | Croatia | Respiratory: Other | 8 | 32 | 64 | >64 | >64 | 0.25 | >16 | 8 | >16 | >32 | >32 |
| 1983237 | *A. baumannii* | 2019 | Greece | Respiratory: Bronchoalveolar lavage | 4 | 64 | >64 | >64 | >64 | 2 | >16 | 4 | >16 | 2 | 32 |
| 2031462 | *A. baumannii* | 2019 | Greece | Cardiovascular: Blood | 8 | 16 | >64 | >64 | >64 | 0.25 | >16 | 8 | 16 | >32 | >32 |
| 1971733 | *A. baumannii* | 2019 | Latvia | Gastrointestinal: Pancreas | 4 | 64 | 64 | >64 | >64 | 0.25 | >16 | 8 | >16 | >32 | 16 |
| 1983241 | *A. baumannii* | 2019 | Greece | Cardiovascular: Blood | 4 | 32 | 64 | >64 | >64 | 1 | >16 | 4 | >16 | >32 | >32 |
| 1971752 | *A. baumannii* | 2019 | Latvia | Gastrointestinal: Pancreas | 4 | 64 | 32 | >64 | >64 | 0.25 | >16 | 8 | >16 | >32 | 32 |

| | | | | | MIC values (µg/mL) | | | | | | | | | | | |
|---|---|---|---|---|---|---|---|---|---|---|---|---|---|---|---|---|
| | | | | **CLSI Cut-off Values (µg/mL)** | N/A | S≤4/8 R≥16/32 | S≤8 R≥32 | S≤8 R≥32 | S≤8 R≥64 | S≤2 R≥8 | S≤4 R≥16 | S≤2 R≥8 | S≤2 R≥8 | S≤4 R≥16 | S≤2/38 R≥4/76 |
| **IHMA Clinical Isolate #** | **Organism** | **Year Collected** | **Country** | **Body Location** | **OMN6** | **SAM (2:1)** | **FEP** | **CAZ** | **CRO** | **COL** | **GEN** | **LVX** | **MEM** | **TET** | **TSX (1:19)** |
| 2031524 | *A. baumannii* | 2019 | Greece | Genitourinary: Urine | 4 | 64 | >64 | >64 | 64 | 0.25 | >16 | 2 | >16 | >32 | 16 |
| 2020360 | *A. baumannii* | 2019 | Croatia | Respiratory: Other | 8 | 16 | 32 | >64 | >64 | 0.25 | >16 | 8 | 16 | >32 | 32 |
| 2031416 | *A. baumannii* | 2019 | Greece | Cardiovascular: Blood | 8 | 32 | 64 | >64 | >64 | 0.25 | 16 | 4 | >16 | >32 | >32 |
| 2020358 | *A. baumannii* | 2019 | Croatia | Respiratory: Other | 4 | 64 | 16 | >64 | >64 | 0.25 | >16 | 2 | >16 | 2 | 4 |
| 2031441 | *A. baumannii* | 2019 | Greece | Cardiovascular: Blood | 8 | 64 | 64 | >64 | >64 | 0.5 | >16 | 32 | >16 | >32 | 1 |
| 2031511 | *A. baumannii* | 2019 | Greece | Genitourinary: Urine | 4 | ≤1 | 0.5 | 2 | 8 | 0.25 | 0.25 | ≤0.12 | ≤0.06 | 0.25 | ≤0.06 |
| 1983235 | *A. baumannii* | 2019 | Greece | Respiratory: Sputum | 8 | 32 | 64 | >64 | >64 | 1 | >16 | 4 | >16 | 4 | 32 |
| 1971673 | *A. baumannii* | 2019 | Latvia | Respiratory: Endotracheal aspirate | 4 | 64 | 64 | >64 | >64 | 0.25 | >16 | 8 | >16 | >32 | >32 |
| 1983243 | *A. baumannii* | 2019 | Greece | Respiratory: Endotracheal aspirate | 4 | 64 | >64 | >64 | >64 | 0.5 | >16 | 8 | >16 | >32 | >32 |
| 2119058 | *A. baumannii* | 2019 | Germany | Gastrointestinal: Large Colon | 8 | ≤1 | 1 | 2 | 8 | 0.25 | 0.5 | ≤0.12 | 0.12 | 0.25 | 0.12 |
| 1954162 | *A. baumannii* | 2019 | Germany | Gastrointestinal: Small Colon | 4 | 2 | 1 | 2 | 8 | 0.25 | 0.5 | ≤0.12 | 0.12 | 0.25 | 0.12 |
| 1983230 | *A. baumannii* | 2019 | Greece | Respiratory: Sputum | 8 | 32 | 32 | >64 | >64 | 0.5 | >16 | 4 | >16 | >32 | >32 |
| 1971821 | *A. baumannii* | 2019 | Latvia | Respiratory: Bronchoalveolar lavage | 8 | 64 | >64 | >64 | >64 | 0.25 | >16 | 2 | >16 | 0.5 | 4 |

| | | | | | MIC values (µg/mL) | | | | | | | | | | |
|---|---|---|---|---|---|---|---|---|---|---|---|---|---|---|---|
| | | | | CLSI Cut-off Values (µg/mL) | N/A | S≤4/8 R≥16/32 | S≤8 R≥32 | S≤8 R≥32 | S≤8 R≥64 | S≤2 R≥8 | S≤4 R≥16 | S≤2 R≥8 | S≤2 R≥8 | S≤4 R≥16 | S≤2/38 R≥4/76 |
| **IHMA Clinical Isolate #** | **Organism** | **Year Collected** | **Country** | **Body Location** | **OMN6** | **SAM (2:1)** | **FEP** | **CAZ** | **CRO** | **COL** | **GEN** | **LVX** | **MEM** | **TET** | **TSX (1:19)** |
| 1974405 | *A. baumannii* | 2019 | Germany | Genitourinary: Other | 4 | ≤1 | ≤0.25 | 1 | 2 | 0.25 | 0.5 | ≤0.12 | ≤0.06 | 0.25 | ≤0.06 |
| 2039140 | *A. baumannii* | 2019 | Hungary | Respiratory: Endotracheal aspirate | 4 | 64 | 16 | >64 | >64 | 0.25 | >16 | 2 | >16 | 1 | 4 |
| 1971746 | *A. baumannii* | 2019 | Latvia | Gastrointestinal: Pancreas | 4 | 64 | 64 | >64 | >64 | 0.25 | >16 | 8 | >16 | >32 | 32 |
| 1989600 | *A. baumannii* | 2019 | Germany | Respiratory: Endotracheal aspirate | 4 | ≤1 | 2 | 4 | 8 | 0.25 | 0.5 | ≤0.12 | 0.25 | 0.25 | 0.12 |
| 2039073 | *A. baumannii* | 2019 | Hungary | Cardiovascular: Blood | 4 | 32 | 32 | >64 | >64 | 0.25 | >16 | 4 | >16 | 4 | 2 |
| 2031449 | *A. baumannii* | 2019 | Greece | Cardiovascular: Blood | 8 | 64 | 64 | >64 | >64 | 0.25 | >16 | 4 | >16 | >32 | >32 |
| 1968762 | *A. baumannii* | 2019 | Lithuania | Respiratory: Bronchoalveolar lavage | 4 | 32 | 16 | >64 | >64 | 0.25 | >16 | 4 | 16 | >32 | 0.5 |
| 2023557 | *A. baumannii* | 2019 | Hungary | Gastrointestinal: Gall Bladder | 4 | 64 | 8 | >64 | >64 | 0.25 | >16 | 4 | >16 | 2 | 1 |
| 1968785 | *A. baumannii* | 2019 | Lithuania | Respiratory: Bronchoalveolar lavage | 8 | 64 | 64 | 32 | >64 | 4 | 16 | 2 | >16 | >32 | 8 |
| 1983236 | *A. baumannii* | 2019 | Greece | Respiratory: Bronchoalveolar lavage | 4 | 64 | 64 | >64 | >64 | 1 | >16 | 8 | >16 | >32 | >32 |
| 1973940 | *A. baumannii* | 2019 | Poland | Respiratory: Endotracheal aspirate | 4 | 8 | 8 | 64 | >64 | 0.25 | 16 | 16 | >16 | >32 | >32 |

| | | | | MIC values (μg/mL) | | | | | | | | | | | |
|---|---|---|---|---|---|---|---|---|---|---|---|---|---|---|---|
| | | | | **CLSI Cut-off Values (μg/mL)** | N/A | S≤4/8 R≥16/32 | S≤8 R≥32 | S≤8 R≥32 | S≤8 R≥64 | S≤2 R≥8 | S≤4 R≥16 | S≤2 R≥8 | S≤2 R≥8 | S≤4 R≥16 | S≤2/38 R≥4/76 |
| **IHMA Clinical Isolate #** | **Organism** | **Year Collected** | **Country** | **Body Location** | **OMN6** | **SAM (2:1)** | **FEP** | **CAZ** | **CRO** | **COL** | **GEN** | **LVX** | **MEM** | **TET** | **TSX (1:19)** |
| 2031461 | *A. baumannii* | 2019 | Greece | Cardiovascular: Blood | 8 | 64 | >64 | >64 | >64 | 1 | >16 | 8 | >16 | >32 | 32 |
| 2039136 | *A. baumannii* | 2019 | Hungary | Respiratory: Endotracheal aspirate | 8 | 32 | 32 | >64 | >64 | 0.25 | >16 | 8 | >16 | >32 | 32 |
| 2038979 | *A. baumannii* | 2019 | Hungary | Bodily Fluids: Peritoneal | 4 | 64 | 32 | >64 | >64 | 0.25 | >16 | 2 | >16 | 2 | 1 |
| 1973923 | *A. baumannii* | 2019 | Poland | Respiratory: Endotracheal aspirate | 4 | 32 | 8 | 64 | >64 | 0.25 | >16 | 2 | 0.25 | >32 | 16 |
| 1961080 | *A. baumannii* | 2019 | Portugal | Cardiovascular: Blood | 4 | 2 | 1 | 2 | 8 | 0.5 | 0.5 | ≤0.12 | 0.12 | 0.5 | ≤0.06 |
| 2078129 | *A. baumannii* | 2019 | Italy | Respiratory: Sputum | 4 | 32 | 64 | >64 | >64 | 0.25 | >16 | >32 | 16 | >32 | 32 |
| 1994284 | *A. baumannii* | 2019 | Italy | Genitourinary: Urine | 4 | 8 | 16 | 64 | >64 | 0.25 | >16 | 16 | 16 | >32 | 32 |
| 2019331 | *A. baumannii* | 2019 | Romania | Respiratory: Endotracheal aspirate | 8 | 32 | 64 | >64 | >64 | 0.25 | >16 | 8 | >16 | >32 | >32 |
| 2019266 | *A. baumannii* | 2019 | Romania | Respiratory: Sputum | 4 | 16 | 32 | >64 | >64 | 0.25 | >16 | 8 | >16 | 0.5 | 1 |
| 2019315 | *A. baumannii* | 2019 | Romania | Cardiovascular: Blood | 4 | 2 | 0.5 | 1 | 4 | 0.25 | 1 | ≤0.12 | ≤0.06 | 0.25 | 0.12 |
| 2030964 | *A. baumannii* | 2019 | Italy | Bodily Fluids: Peritoneal | 4 | 64 | 64 | >64 | >64 | ≤0.12 | 0.5 | 2 | >16 | >32 | 0.5 |
| 1983210 | *A. baumannii* | 2019 | Greece | Respiratory: Bronchial brushing | 4 | 32 | 32 | >64 | >64 | >8 | >16 | 8 | >16 | 4 | 32 |

| | | | | | | MIC values (µg/mL) | | | | | | | | | |
|---|---|---|---|---|---|---|---|---|---|---|---|---|---|---|---|
| | | | | **CLSI Cut-off Values (µg/mL)** | N/A | S≤4/8 R≥16/32 | S≤8 R≥32 | S≤8 R≥32 | S≤8 R≥64 | S≤2 R≥8 | S≤4 R≥16 | S≤2 R≥8 | S≤2 R≥8 | S≤4 R≥16 | S≤2/38 R≥4/76 |
| **IHMA Clinical Isolate #** | **Organism** | **Year Collected** | **Country** | **Body Location** | **OMN6** | **SAM (2:1)** | **FEP** | **CAZ** | **CRO** | **COL** | **GEN** | **LVX** | **MEM** | **TET** | **TSX (1:19)** |
| 2078186 | *A. baumannii* | 2019 | Italy | Respiratory: Sputum | 4 | 16 | 32 | 64 | >64 | 0.25 | >16 | >32 | >16 | >32 | >32 |
| 2078054 | *A. baumannii* | 2019 | Italy | Respiratory: Sputum | 4 | 32 | 32 | 64 | >64 | 0.25 | >16 | >32 | 16 | >32 | 32 |
| 1994130 | *A. baumannii* | 2019 | Italy | Respiratory: Sputum | 8 | 8 | 16 | >64 | >64 | >8 | >16 | >32 | 16 | 8 | 32 |
| 1971730 | *A. baumannii* | 2019 | Latvia | Gastrointestinal: Stomach | 8 | 32 | >64 | >64 | >64 | 0.25 | >16 | 2 | >16 | 0.5 | 4 |
| 1983240 | *A. baumannii* | 2019 | Greece | Respiratory: Bronchoalveolar lavage | 4 | 32 | 16 | >64 | >64 | 0.25 | >16 | 32 | 16 | >32 | 1 |
| 2031456 | *A. baumannii* | 2019 | Greece | Cardiovascular: Blood | 4 | 32 | 32 | >64 | >64 | 0.25 | >16 | 32 | >16 | >32 | >32 |
| 1984552 | *A. baumannii* | 2019 | Lithuania | Genitourinary: Urine | 4 | 64 | 64 | >64 | >64 | 0.25 | >16 | 8 | >16 | >32 | 2 |
| 2051395 | *A. baumannii* | 2019 | Serbia | Cardiovascular: Blood | 4 | 64 | 64 | >64 | >64 | 0.25 | >16 | 8 | >16 | 32 | 8 |
| 1952420 | *A. baumannii* | 2019 | Italy | Bodily Fluids: Peritoneal | 4 | 16 | 16 | 32 | >64 | 0.25 | >16 | >32 | 16 | >32 | 32 |
| 2051484 | *A. baumannii* | 2019 | Serbia | Respiratory: Endotracheal aspirate | 4 | 16 | 16 | >64 | >64 | 0.25 | >16 | 4 | >16 | >32 | 32 |
| 1968809 | *A. baumannii* | 2019 | Lithuania | Respiratory: Bronchoalveolar lavage | 4 | 64 | 64 | >64 | >64 | 1 | >16 | 4 | >16 | >32 | >32 |
| 1983229 | *A. baumannii* | 2019 | Greece | Cardiovascular: Blood | 4 | 64 | 64 | >64 | >64 | 1 | >16 | 8 | 16 | 4 | >32 |

| | | | | CLSI Cut-off Values (µg/mL) | N/A | S≤4/8 R≥16/32 | S≤8 R≥32 | S≤8 R≥32 | S≤8 R≥64 | S≤2 R≥8 | S≤4 R≥16 | S≤2 R≥8 | S≤2 R≥8 | S≤4 R≥16 | S≤2/38 R≥4/76 |
|---|---|---|---|---|---|---|---|---|---|---|---|---|---|---|---|
| | | | | | MIC values (µg/mL) | | | | | | | | | | |
| **IHMA Clinical Isolate #** | **Organism** | **Year Collected** | **Country** | **Body Location** | **OMN6** | **SAM (2:1)** | **FEP** | **CAZ** | **CRO** | **COL** | **GEN** | **LVX** | **MEM** | **TET** | **TSX (1:19)** |
| 1984668 | *A. baumannii* | 2019 | Lithuania | Respiratory: Bronchoalveolar lavage | 8 | 64 | 64 | >64 | >64 | 0.25 | >16 | 8 | >16 | >32 | 1 |
| 1971652 | *A. baumannii* | 2019 | Latvia | Cardiovascular: Blood | 8 | 16 | 32 | 16 | >64 | 0.25 | >16 | 2 | >16 | 1 | 0.12 |
| 2035039 | *A. baumannii* | 2019 | Serbia | Cardiovascular: Blood | 8 | 32 | 64 | >64 | >64 | 0.25 | >16 | 16 | >16 | >32 | >32 |
| 1984695 | *A. baumannii* | 2019 | Lithuania | Respiratory: Bronchoalveolar lavage | 4 | 8 | 8 | >64 | >64 | 0.25 | 0.5 | 2 | >16 | >32 | 16 |
| 1968732 | *A. baumannii* | 2019 | Lithuania | Respiratory: Bronchoalveolar lavage | 4 | 64 | 64 | >64 | >64 | 0.25 | >16 | 8 | >16 | >32 | 1 |
| 1971717 | *A. baumannii* | 2019 | Latvia | Respiratory: Bronchoalveolar lavage | 8 | 32 | >64 | >64 | >64 | 0.25 | 2 | 2 | >16 | 1 | 8 |
| 2051611 | *A. baumannii* | 2019 | Serbia | Respiratory: Endotracheal aspirate | 4 | 32 | >64 | >64 | >64 | 0.25 | >16 | 8 | >16 | >32 | >32 |
| 1983199 | *A. baumannii* | 2019 | Greece | Genitourinary: Urine | 4 | 32 | 64 | >64 | >64 | 0.25 | >16 | 8 | >16 | >32 | >32 |
| 2051610 | *A. baumannii* | 2019 | Serbia | Cardiovascular: Blood | 4 | 16 | 8 | >64 | >64 | ≤0.12 | 1 | 32 | >16 | 1 | 8 |
| 1971808 | *A. baumannii* | 2019 | Latvia | Respiratory: Sputum | 8 | 16 | 32 | 16 | >64 | 0.25 | 1 | 2 | >16 | 1 | 0.12 |
| 2051433 | *A. baumannii* | 2019 | Serbia | Gastrointestinal: Liver | 4 | 16 | 8 | 64 | >64 | 0.25 | >16 | 2 | >16 | >32 | 1 |

| | | | | | MIC values (µg/mL) | | | | | | | | | | | |
|---|---|---|---|---|---|---|---|---|---|---|---|---|---|---|---|---|
| | | | | **CLSI Cut-off Values (µg/mL)** | N/A | S≤4/8 R≥16/32 | S≤8 R≥32 | S≤8 R≥32 | S≤8 R≥64 | S≤2 R≥8 | S≤4 R≥16 | S≤2 R≥8 | S≤2 R≥8 | S≤4 R≥16 | S≤2/38 R≥4/76 |
| **IHMA Clinical Isolate #** | **Organism** | **Year Collected** | **Country** | **Body Location** | **OMN6** | **SAM (2:1)** | **FEP** | **CAZ** | **CRO** | **COL** | **GEN** | **LVX** | **MEM** | **TET** | **TSX (1:19)** |
| 2039139 | *A. baumannii* | 2019 | Hungary | Respiratory: Endotracheal aspirate | 4 | 32 | 16 | >64 | >64 | 0.25 | >16 | 4 | >16 | 2 | 1 |
| 1971810 | *A. baumannii* | 2019 | Latvia | Respiratory: Bronchoalveolar lavage | 8 | 16 | 32 | 16 | >64 | 0.25 | >16 | 2 | >16 | 0.5 | 0.12 |
| 2058360 | *A. baumannii* | 2019 | Slovenia | Genitourinary: Urine | 8 | 4 | 4 | 16 | >64 | 0.25 | >16 | 1 | 0.5 | 1 | 0.12 |
| 1984733 | *A. baumannii* | 2019 | Lithuania | Cardiovascular: Blood | 4 | 64 | 64 | >64 | >64 | 0.25 | >16 | 4 | >16 | >32 | 2 |
| 2058247 | *A. baumannii* | 2019 | Slovenia | Respiratory: Bronchoalveolar lavage | 4 | ≤1 | 1 | 2 | 8 | 0.25 | 0.25 | ≤0.12 | ≤0.06 | 0.25 | 0.12 |
| 1967860 | *A. baumannii* | 2019 | Spain | Cardiovascular: Blood | 8 | 2 | 4 | 2 | 8 | 0.25 | 4 | ≤0.12 | 0.12 | >32 | 0.25 |
| 2039121 | *A. baumannii* | 2019 | Hungary | Respiratory: Endotracheal aspirate | 8 | 64 | 8 | >64 | >64 | 0.25 | >16 | 8 | 8 | 2 | 16 |
| 2007374 | *A. baumannii* | 2019 | Spain | Respiratory: Sputum | 4 | 32 | 32 | 8 | 32 | 0.25 | 0.5 | 16 | >16 | >32 | 2 |
| 2014048 | *A. baumannii* | 2019 | Sweden | Gastrointestinal: Stomach | 8 | 8 | 8 | >64 | >64 | >8 | >16 | >32 | 16 | 8 | 32 |
| 1984688 | *A. baumannii* | 2019 | Lithuania | Respiratory: Bronchoalveolar lavage | 4 | 64 | 64 | >64 | >64 | 0.25 | >16 | 8 | >16 | >32 | 1 |
| 2039155 | *A. baumannii* | 2019 | Hungary | Respiratory: Bronchoalveolar lavage | 4 | 32 | 32 | >64 | >64 | 0.25 | >16 | 16 | 16 | >32 | 32 |

| | | | | | MIC values (μg/mL) | | | | | | | | | | |
|---|---|---|---|---|---|---|---|---|---|---|---|---|---|---|---|
| | | | | **CLSI Cut-off Values (μg/mL)** | N/A | S≤4/8 R≥16/32 | S≤8 R≥32 | S≤8 R≥32 | S≤8 R≥64 | S≤2 R≥8 | S≤4 R≥16 | S≤2 R≥8 | S≤2 R≥8 | S≤4 R≥16 | S≤2/38 R≥4/76 |
| **IHMA Clinical Isolate #** | **Organism** | **Year Collected** | **Country** | **Body Location** | **OMN6** | **SAM (2:1)** | **FEP** | **CAZ** | **CRO** | **COL** | **GEN** | **LVX** | **MEM** | **TET** | **TSX (1:19)** |
| 1984669 | *A. baumannii* | 2019 | Lithuania | Respiratory: Endotracheal aspirate | 4 | 32 | 64 | >64 | >64 | 0.25 | >16 | 8 | >16 | >32 | 0.5 |
| 1973792 | *A. baumannii* | 2019 | Poland | Cardiovascular: Blood | 8 | 8 | 8 | 64 | >64 | 0.25 | 16 | 16 | >16 | >32 | >32 |
| 2108908 | *A. baumannii* | 2019 | Italy | Gastrointestinal: Abscess | 4 | 4 | 8 | 64 | >64 | ≤0.12 | >16 | 16 | 8 | ≤0.12 | 4 |
| 2078203 | *A. baumannii* | 2019 | Italy | Cardiovascular: Blood | 4 | 32 | 64 | 64 | >64 | 0.5 | >16 | 16 | >16 | >32 | 16 |
| 1971626 | *A. baumannii* | 2019 | Latvia | Cardiovascular: Blood | 4 | 16 | 32 | 16 | >64 | 0.25 | >16 | 2 | >16 | 1 | 0.12 |
| 2003382 | *A. baumannii* | 2019 | Turkey | Cardiovascular: Blood | 4 | 4 | 8 | 8 | >64 | 0.25 | >16 | 4 | 0.5 | 1 | 0.12 |
| 1971716 | *A. baumannii* | 2019 | Latvia | Respiratory: Sputum | 8 | 32 | >64 | >64 | >64 | 0.25 | 1 | 4 | >16 | 0.5 | 4 |
| 1984758 | *A. baumannii* | 2019 | Lithuania | Cardiovascular: Blood | 4 | 4 | 4 | 16 | >64 | ≤0.12 | >16 | 0.5 | 0.5 | >32 | 8 |
| 1963810 | *A. baumannii* | 2019 | Turkey | Genitourinary: Urine | 4 | 32 | 32 | 32 | >64 | 0.25 | 2 | >32 | 16 | >32 | >32 |
| 1957343 | *A. baumannii* | 2019 | Portugal | Gastrointestinal: Gall Bladder | 4 | 2 | 2 | 4 | 8 | ≤0.12 | 0.5 | ≤0.12 | 0.12 | 0.5 | 0.12 |
| 1993625 | *A. baumannii* | 2019 | Turkey | Respiratory: Endotracheal aspirate | 8 | 4 | 32 | >64 | >64 | 0.25 | >16 | 32 | >16 | >32 | >32 |
| 1963865 | *A. baumannii* | 2019 | Turkey | Bodily Fluids: Thoracentesis | 4 | 32 | 32 | 32 | >64 | 0.5 | 4 | >32 | 16 | >32 | >32 |
| 1984666 | *A. baumannii* | 2019 | Lithuania | Respiratory: Endotracheal aspirate | 8 | 64 | 64 | >64 | >64 | 0.5 | >16 | 8 | >16 | >32 | 1 |

| | | | | MIC values (µg/mL) | | | | | | | | | | | |
|---|---|---|---|---|---|---|---|---|---|---|---|---|---|---|---|
| | | | | **CLSI Cut-off Values (µg/mL)** | N/A | S≤4/8 R≥16/32 | S≤8 R≥32 | S≤8 R≥32 | S≤8 R≥64 | S≤2 R≥8 | S≤4 R≥16 | S≤2 R≥8 | S≤2 R≥8 | S≤4 R≥16 | S≤2/38 R≥4/76 |
| **IHMA Clinical Isolate #** | **Organism** | **Year Collected** | **Country** | **Body Location** | **OMN6** | **SAM (2:1)** | **FEP** | **CAZ** | **CRO** | **COL** | **GEN** | **LVX** | **MEM** | **TET** | **TSX (1:19)** |
| 1984671 | *A. baumannii* | 2019 | Lithuania | Respiratory: Bronchial brushing | 8 | 64 | 64 | >64 | >64 | 0.25 | >16 | 8 | >16 | >32 | 1 |
| 2003388 | *A. baumannii* | 2019 | Turkey | Cardiovascular: Blood | 4 | 4 | 8 | 8 | >64 | 0.25 | >16 | 4 | 0.5 | 1 | 0.12 |
| 2003373 | *A. baumannii* | 2019 | Turkey | Cardiovascular: Blood | 4 | 2 | 1 | 2 | 8 | 0.25 | 0.25 | ≤0.12 | 0.12 | 0.5 | ≤0.06 |
| 1993618 | *A. baumannii* | 2019 | Turkey | Respiratory: Bronchoalveolar lavage | 4 | 32 | 32 | >64 | >64 | 0.5 | >16 | 32 | >16 | >32 | >32 |
| 1973996 | *A. baumannii* | 2019 | Poland | Respiratory: Endotracheal aspirate | 4 | 2 | 4 | 4 | 16 | 0.25 | 0.5 | ≤0.12 | 0.12 | 0.5 | 0.5 |
| 1993626 | *A. baumannii* | 2019 | Turkey | Respiratory: Sputum | 8 | >64 | 64 | >64 | >64 | 0.25 | >16 | 8 | >16 | >32 | 1 |
| 2066250 | *A. baumannii* | 2019 | Poland | Bodily Fluids: Peritoneal | 8 | 8 | 16 | >64 | >64 | 0.25 | 2 | 8 | >16 | >32 | >32 |
| 2035030 | *A. baumannii* | 2019 | Serbia | Respiratory: Endotracheal aspirate | 8 | 16 | 32 | >64 | >64 | 0.25 | >16 | 8 | >16 | >32 | >32 |
| 2035026 | *A. baumannii* | 2019 | Serbia | Respiratory: Endotracheal aspirate | 4 | 32 | 16 | >64 | >64 | 0.25 | >16 | 4 | >16 | >32 | >32 |
| 1993617 | *A. baumannii* | 2019 | Turkey | Respiratory: Endotracheal aspirate | 4 | 8 | 16 | 32 | >64 | >8 | >16 | 16 | 16 | >32 | 32 |
| 2003371 | *A. baumannii* | 2019 | Turkey | Cardiovascular: Blood | 4 | 64 | 32 | >64 | >64 | 0.25 | 0.25 | 16 | >16 | >32 | >32 |

| | | | | MIC values (µg/mL) | | | | | | | | | | | |
|---|---|---|---|---|---|---|---|---|---|---|---|---|---|---|---|
| | | | | **CLSI Cut-off Values (µg/mL)** | N/A | S≤4/8 R≥16/32 | S≤8 R≥32 | S≤8 R≥32 | S≤8 R≥64 | S≤2 R≥8 | S≤4 R≥16 | S≤2 R≥8 | S≤2 R≥8 | S≤4 R≥16 | S≤2/38 R≥4/76 |
| **IHMA Clinical Isolate #** | **Organism** | **Year Collected** | **Country** | **Body Location** | **OMN6** | **SAM (2:1)** | **FEP** | **CAZ** | **CRO** | **COL** | **GEN** | **LVX** | **MEM** | **TET** | **TSX (1:19)** |
| 1973970 | *A. baumannii* | 2019 | Poland | Respiratory: Endotracheal aspirate | 4 | 8 | 16 | 64 | >64 | 2 | 0.25 | 4 | >16 | >32 | >32 |
| 2035464 | *A. baumannii* | 2019 | Ukraine | Respiratory: Sputum | 4 | 32 | 64 | 16 | >64 | 0.25 | >16 | 4 | 16 | 4 | 1 |
| 2019236 | *A. baumannii* | 2019 | Romania | Cardiovascular: Blood | 8 | 64 | 32 | >64 | >64 | 0.25 | >16 | 4 | 1 | >32 | >32 |
| 2035089 | *A. baumannii* | 2019 | Serbia | Respiratory: Endotracheal aspirate | 8 | 32 | 32 | >64 | >64 | 0.25 | >16 | 8 | >16 | >32 | >32 |
| 2118005 | *A. baumannii* | 2019 | Russia | Gastrointestinal: Large Colon | 4 | 2 | 1 | 2 | 8 | 0.25 | 0.25 | ≤0.12 | 0.12 | 0.25 | 0.12 |
| 2035500 | *A. baumannii* | 2019 | Ukraine | Cardiovascular: Blood | 4 | 4 | 8 | 32 | >64 | 0.25 | 4 | 8 | 8 | 1 | 4 |
| 2118004 | *A. baumannii* | 2019 | Russia | Gastrointestinal: Large Colon | 8 | 8 | 4 | 32 | >64 | 0.25 | 4 | 32 | 16 | 2 | 4 |
| 2029125 | *A. baumannii* | 2019 | Ukraine | Cardiovascular: Blood | 8 | 4 | 4 | 16 | >64 | 1 | 0.25 | 4 | 0.5 | 1 | 4 |
| 1973963 | *A. baumannii* | 2019 | Poland | Respiratory: Endotracheal aspirate | 4 | 16 | 32 | >64 | >64 | 0.25 | 0.5 | 4 | 2 | >32 | >32 |
| 2060117 | *A. baumannii* | 2019 | Russia | Gastrointestinal: Abscess | 4 | >64 | >64 | >64 | >64 | 0.25 | >16 | 8 | >16 | 2 | 32 |
| 2035090 | *A. baumannii* | 2019 | Serbia | Respiratory: Endotracheal aspirate | 8 | 16 | 32 | >64 | >64 | 0.25 | >16 | 8 | >16 | >32 | >32 |
| 2051501 | *A. baumannii* | 2019 | Serbia | Genitourinary: Urine | 8 | 16 | 16 | >64 | >64 | 0.25 | >16 | 8 | 16 | >32 | >32 |

| | | | | | MIC values (µg/mL) | | | | | | | | | | |
|---|---|---|---|---|---|---|---|---|---|---|---|---|---|---|---|
| | | | | CLSI Cut-off Values (µg/mL) | N/A | S≤4/8 R≥16/32 | S≤8 R≥32 | S≤8 R≥32 | S≤8 R≥64 | S≤2 R≥8 | S≤4 R≥16 | S≤2 R≥8 | S≤2 R≥8 | S≤4 R≥16 | S≤2/38 R≥4/76 |
| **IHMA Clinical Isolate #** | **Organism** | **Year Collected** | **Country** | **Body Location** | **OMN6** | **SAM (2:1)** | **FEP** | **CAZ** | **CRO** | **COL** | **GEN** | **LVX** | **MEM** | **TET** | **TSX (1:19)** |
| 2051527 | *A. baumannii* | 2019 | Serbia | Respiratory: Endotracheal aspirate | 4 | 16 | 8 | 64 | >64 | 0.25 | 0.25 | 2 | >16 | >32 | 0.5 |
| 2058329 | *A. baumannii* | 2019 | Slovenia | Cardiovascular: Blood | 8 | 2 | 1 | 2 | 8 | 0.25 | 0.5 | ≤0.12 | ≤0.06 | 0.5 | ≤0.06 |
| 1957424 | *A. baumannii* | 2019 | Portugal | Cardiovascular: Blood | 4 | 16 | 16 | 64 | >64 | 0.25 | >16 | 32 | 16 | >32 | 32 |
| 2035052 | *A. baumannii* | 2019 | Serbia | Bodily Fluids: Peritoneal | 8 | 32 | 32 | >64 | >64 | 0.5 | >16 | 16 | >16 | >32 | 32 |
| 1957322 | *A. baumannii* | 2019 | Portugal | Genitourinary: Urine | 4 | 8 | 4 | 1 | 4 | 0.25 | 1 | 32 | 8 | >32 | 0.5 |
| 2019421 | *A. baumannii* | 2019 | Romania | Cardiovascular: Blood | 4 | 8 | 32 | >64 | >64 | 0.25 | 1 | 4 | 16 | 4 | 0.12 |
| 2003517 | *A. baumannii* | 2019 | Turkey | Respiratory: Endotracheal aspirate | 4 | >64 | >64 | >64 | >64 | 0.25 | >16 | 16 | >16 | >32 | 32 |
| 1970734 | *A. baumannii* | 2019 | Switzerland | Respiratory: Sputum | 4 | >64 | >64 | >64 | >64 | 0.25 | >16 | 32 | >16 | 8 | 0.5 |
| 2062415 | *A. baumannii* | 2019 | Russia | Bodily Fluids: Peritoneal | 4 | 64 | >64 | >64 | >64 | ≤0.12 | 8 | >32 | >16 | >32 | >32 |
| 2035064 | *A. baumannii* | 2019 | Serbia | Cardiovascular: Blood | 8 | 32 | 32 | >64 | >64 | 0.25 | >16 | 8 | >16 | >32 | >32 |
| 1993813 | *A. baumannii* | 2019 | Turkey | Cardiovascular: Blood | 4 | >64 | 32 | 64 | >64 | 0.25 | ≤0.12 | 16 | >16 | >32 | >32 |
| 2009420 | *A. baumannii* | 2019 | Turkey | Gastrointestinal: Other | 4 | 64 | 64 | >64 | >64 | ≤0.12 | 0.25 | 16 | >16 | >32 | >32 |
| 1993762 | *A. baumannii* | 2019 | Turkey | Bodily Fluids: Abscess / Pus | 8 | >64 | >64 | >64 | >64 | 1 | 0.25 | 16 | >16 | >32 | >32 |

| | | | | | MIC values (µg/mL) | | | | | | | | | | |
|---|---|---|---|---|---|---|---|---|---|---|---|---|---|---|---|
| | | | | **CLSI Cut-off Values (µg/mL)** | N/A | S≤4/8 R≥16/32 | S≤8 R≥32 | S≤8 R≥32 | S≤8 R≥64 | S≤2 R≥8 | S≤4 R≥16 | S≤2 R≥8 | S≤2 R≥8 | S≤4 R≥16 | S≤2/38 R≥4/76 |
| **IHMA Clinical Isolate #** | **Organism** | **Year Collected** | **Country** | **Body Location** | **OMN6** | **SAM (2:1)** | **FEP** | **CAZ** | **CRO** | **COL** | **GEN** | **LVX** | **MEM** | **TET** | **TSX (1:19)** |
| 2051483 | *A. baumannii* | 2019 | Serbia | Cardiovascular: Blood | 4 | 32 | 16 | >64 | >64 | 0.25 | >16 | 4 | >16 | 4 | 8 |
| 1986579 | *A. baumannii* | 2019 | Turkey | Respiratory: Endotracheal aspirate | 4 | 64 | >64 | >64 | >64 | 0.25 | >16 | 8 | 8 | 2 | 32 |
| 1963864 | *A. baumannii* | 2019 | Turkey | Respiratory: Lungs | 4 | 64 | 32 | 64 | >64 | 0.5 | 2 | >32 | 16 | >32 | >32 |
| 2051432 | *A. baumannii* | 2019 | Serbia | Gastrointestinal: Stomach | 4 | 32 | 32 | >64 | >64 | 0.25 | >16 | 8 | >16 | >32 | 1 |
| 2003491 | *A. baumannii* | 2019 | Turkey | Cardiovascular: Blood | 4 | 32 | 64 | >64 | >64 | 0.25 | 8 | 16 | >16 | >32 | >32 |
| 2051455 | *A. baumannii* | 2019 | Serbia | Genitourinary: Urine | 4 | 32 | 64 | 64 | >64 | 0.25 | >16 | 8 | >16 | 8 | 32 |
| 1968017 | *A. baumannii* | 2019 | Spain | Respiratory: Endotracheal aspirate | 4 | 2 | 1 | 2 | 8 | 0.25 | 0.5 | ≤0.12 | ≤0.06 | 0.5 | ≤0.06 |
| 2081197 | *A. baumannii* | 2019 | Spain | Gastrointestinal: Other | 4 | 16 | 16 | 4 | 32 | 1 | >16 | 32 | >16 | >32 | 1 |
| 2003464 | *A. baumannii* | 2019 | Turkey | Cardiovascular: Blood | 8 | 32 | 32 | >64 | >64 | 0.5 | >16 | 8 | >16 | >32 | 2 |
| 2023039 | *A. baumannii* | 2019 | United Kingdom | Bodily Fluids: Peritoneal | 8 | ≤1 | 1 | 2 | 8 | 0.25 | 0.5 | ≤0.12 | ≤0.06 | 0.25 | 0.12 |
| 2003503 | *A. baumannii* | 2019 | Turkey | Gastrointestinal: Other | 4 | 16 | 64 | >64 | >64 | 0.25 | 2 | 8 | 16 | >32 | >32 |
| 1987322 | *A. baumannii* | 2019 | Georgia | Respiratory: Endotracheal aspirate | 4 | 16 | 64 | >64 | >64 | ≤0.12 | ≤0.12 | 8 | >16 | >32 | 8 |
| 1963731 | *A. baumannii* | 2019 | Turkey | Genitourinary: Urine | 8 | 16 | 16 | >64 | >64 | >8 | >16 | >32 | >16 | >32 | >32 |

| | | | | MIC values (µg/mL) | | | | | | | | | | | |
|---|---|---|---|---|---|---|---|---|---|---|---|---|---|---|---|
| | | | | **CLSI Cut-off Values (µg/mL)** | N/A | S≤4/8 R≥16/32 | S≤8 R≥32 | S≤8 R≥32 | S≤8 R≥64 | S≤2 R≥8 | S≤4 R≥16 | S≤2 R≥8 | S≤2 R≥8 | S≤4 R≥16 | S≤2/38 R≥4/76 |
| **IHMA Clinical Isolate #** | **Organism** | **Year Collected** | **Country** | **Body Location** | **OMN6** | **SAM (2:1)** | **FEP** | **CAZ** | **CRO** | **COL** | **GEN** | **LVX** | **MEM** | **TET** | **TSX (1:19)** |
| 1986725 | *A. baumannii* | 2019 | Turkey | Cardiovascular: Blood | 4 | 2 | 2 | 4 | 16 | 0.25 | 0.25 | ≤0.12 | ≤0.06 | 0.5 | 0.12 |
| 1975034 | *A. baumannii* | 2019 | Israel | Cardiovascular: Blood | 4 | 32 | 32 | 64 | >64 | 0.25 | >16 | 2 | >16 | 2 | 32 |
| 1993627 | *A. baumannii* | 2019 | Turkey | Respiratory: Sputum | 8 | 64 | 32 | >64 | >64 | 2 | >16 | 8 | >16 | >32 | 32 |
| 1986635 | *A. baumannii* | 2019 | Turkey | Respiratory: Endotracheal aspirate | 8 | 2 | 2 | 4 | 16 | 0.25 | 0.25 | ≤0.12 | 0.12 | 0.5 | 0.12 |
| 2003318 | *A. baumannii* | 2019 | Turkey | Respiratory: Endotracheal aspirate | 4 | >64 | >64 | >64 | >64 | 0.25 | >16 | 8 | >16 | >32 | 32 |
| 2035431 | *A. baumannii* | 2019 | Ukraine | Respiratory: Sputum | 8 | >64 | >64 | 32 | >64 | 0.25 | >16 | 8 | >16 | 2 | 1 |
| 1975151 | *A. baumannii* | 2019 | Israel | Respiratory: Sputum | 8 | 4 | 2 | 2 | 8 | 0.5 | >16 | ≤0.12 | 0.25 | 1 | ≤0.06 |
| 2029115 | *A. baumannii* | 2019 | Ukraine | Bodily Fluids: Abscess / Pus | 8 | 32 | >64 | >64 | >64 | 0.25 | >16 | 8 | 4 | 2 | >32 |
| 1993619 | *A. baumannii* | 2019 | Turkey | Respiratory: Sputum | 4 | 32 | 32 | >64 | >64 | 0.25 | >16 | 8 | >16 | >32 | >32 |
| 2035340 | *A. baumannii* | 2019 | Ukraine | Genitourinary: Urine | 4 | 2 | 2 | 2 | 16 | 0.25 | 0.25 | ≤0.12 | 0.25 | 1 | 0.12 |
| 2035401 | *A. baumannii* | 2019 | Ukraine | Respiratory: Endotracheal aspirate | 4 | 64 | >64 | 64 | >64 | 0.25 | >16 | 8 | 8 | 0.5 | 4 |
| 2003279 | *A. baumannii* | 2019 | Turkey | Genitourinary: Urine | 8 | 64 | 64 | >64 | >64 | 0.25 | >16 | 4 | >16 | >32 | 32 |

| | | | | MIC values (µg/mL) | | | | | | | | | | | |
|---|---|---|---|---|---|---|---|---|---|---|---|---|---|---|---|
| | | | | **CLSI Cut-off Values (µg/mL)** | N/A | S≤4/8 R≥16/32 | S≤8 R≥32 | S≤8 R≥32 | S≤8 R≥64 | S≤2 R≥8 | S≤4 R≥16 | S≤2 R≥8 | S≤2 R≥8 | S≤4 R≥16 | S≤2/38 R≥4/76 |
| **IHMA Clinical Isolate #** | **Organism** | **Year Collected** | **Country** | **Body Location** | **OMN6** | **SAM (2:1)** | **FEP** | **CAZ** | **CRO** | **COL** | **GEN** | **LVX** | **MEM** | **TET** | **TSX (1:19)** |
| 2020256 | *A. baumannii* | 2019 | Israel | Respiratory: Endotracheal aspirate | 4 | 2 | 0.5 | 1 | 1 | 0.25 | 0.25 | ≤0.12 | ≤0.06 | ≤0.12 | 0.25 |
| 2029130 | *A. baumannii* | 2019 | Ukraine | Cardiovascular: Blood | 8 | 32 | >64 | >64 | >64 | 0.25 | >16 | 8 | 4 | 1 | >32 |
| 1963207 | *A. baumannii* | 2019 | Israel | Respiratory: Sputum | 4 | 4 | 4 | 4 | 32 | ≤0.12 | >16 | 4 | 2 | >32 | >32 |
| 1986495 | *A. baumannii* | 2019 | Turkey | Respiratory: Endotracheal aspirate | 4 | ≤1 | 0.5 | 1 | 4 | 0.5 | 0.25 | ≤0.12 | ≤0.06 | 0.25 | ≤0.06 |
| 2029235 | *A. baumannii* | 2019 | Ukraine | Respiratory: Sputum | 4 | 4 | 16 | >64 | >64 | 0.25 | 1 | >32 | 0.5 | 4 | 1 |
| 1969114 | *A. baumannii* | 2019 | Israel | Respiratory: Bronchoalveolar lavage | 8 | 2 | 1 | 2 | 8 | 0.25 | 0.5 | ≤0.12 | 0.12 | 0.5 | 0.12 |
| 1968966 | *A. baumannii* | 2019 | Israel | Respiratory: Sputum | 8 | 8 | 64 | >64 | >64 | 0.25 | 8 | 4 | 16 | >32 | >32 |
| 1993622 | *A. baumannii* | 2019 | Turkey | Respiratory: Sputum | 8 | 16 | 32 | 64 | >64 | 0.25 | 0.5 | 32 | >16 | >32 | >32 |
| 2029141 | *A. baumannii* | 2019 | Ukraine | Cardiovascular: Blood | 4 | 32 | 64 | >64 | >64 | 0.25 | 1 | >32 | >16 | 4 | 1 |
| 1969318 | *A. baumannii* | 2019 | United Kingdom | Respiratory: Sputum | 4 | 64 | 64 | >64 | >64 | 0.25 | >16 | 16 | >16 | >32 | 32 |
| 1958932 | *A. baumannii* | 2019 | United Kingdom | Gastrointestinal: Other | 4 | ≤1 | 1 | 1 | 8 | ≤0.12 | 0.5 | ≤0.12 | 0.12 | 1 | 0.12 |
| 2029201 | *A. baumannii* | 2019 | Ukraine | Bodily Fluids: Abscess / Pus | 8 | 4 | 4 | 16 | >64 | 2 | 0.25 | 4 | 0.25 | 1 | 8 |

| | | | | MIC values (µg/mL) | | | | | | | | | | | |
|---|---|---|---|---|---|---|---|---|---|---|---|---|---|---|---|
| | | | | **CLSI Cut-off Values (µg/mL)** | N/A | S≤4/8 R≥16/32 | S≤8 R≥32 | S≤8 R≥32 | S≤8 R≥64 | S≤2 R≥8 | S≤4 R≥16 | S≤2 R≥8 | S≤2 R≥8 | S≤4 R≥16 | S≤2/38 R≥4/76 |
| **IHMA Clinical Isolate #** | **Organism** | **Year Collected** | **Country** | **Body Location** | **OMN6** | **SAM (2:1)** | **FEP** | **CAZ** | **CRO** | **COL** | **GEN** | **LVX** | **MEM** | **TET** | **TSX (1:19)** |
| 2029860 | *A. baumannii* | 2019 | United Kingdom | Respiratory: Endotracheal aspirate | 4 | ≤1 | ≤0.25 | 1 | 2 | 0.25 | 0.25 | ≤0.12 | ≤0.06 | 0.25 | ≤0.06 |
| 2030102 | *A. baumannii* | 2019 | United Kingdom | Respiratory: Sputum | 8 | 64 | 64 | >64 | >64 | 0.5 | >16 | 8 | >16 | >32 | 2 |
| 2028775 | *A. baumannii* | 2019 | United Kingdom | Respiratory: Endotracheal aspirate | 8 | 8 | 16 | >64 | >64 | 0.25 | 1 | 4 | 4 | 2 | 32 |
| 1987384 | *A. baumannii* | 2019 | Georgia | Respiratory: Endotracheal aspirate | 8 | 64 | 64 | >64 | >64 | 0.25 | >16 | 32 | >16 | >32 | 1 |
| 1974979 | *A. baumannii* | 2019 | Israel | Respiratory: Sputum | 8 | 32 | 64 | >64 | >64 | 0.25 | 16 | 4 | >16 | >32 | 32 |
| 1985503 | *A. baumannii* | 2019 | Venezuela | Bodily Fluids: Abscess / Pus | 4 | 64 | >64 | >64 | >64 | 0.25 | >16 | 8 | >16 | 4 | 0.25 |
| 2029216 | *A. baumannii* | 2019 | Ukraine | Respiratory: Endotracheal aspirate | 4 | 4 | 4 | 16 | >64 | 2 | 0.25 | 4 | 0.25 | 1 | 4 |
| 1975232 | *A. baumannii* | 2019 | Israel | Respiratory: Sputum | 4 | ≤1 | 8 | 4 | 16 | 0.25 | 2 | ≤0.12 | 0.12 | 0.5 | 2 |
| 1975222 | *A. baumannii* | 2019 | Israel | Respiratory: Sputum | 4 | ≤1 | ≤0.25 | 1 | 4 | 0.25 | 0.25 | ≤0.12 | ≤0.06 | 0.25 | 0.12 |
| 2020245 | *A. baumannii* | 2019 | Israel | Respiratory: Endotracheal aspirate | 4 | 2 | 2 | 4 | 8 | 0.25 | >16 | 1 | 0.12 | 1 | 4 |
| 2020241 | *A. baumannii* | 2019 | Israel | Respiratory: Endotracheal aspirate | 4 | 4 | 4 | 4 | 16 | ≤0.12 | 0.5 | ≤0.12 | 0.5 | 1 | 2 |

| | | | | | MIC values (μg/mL) | | | | | | | | | | |
|---|---|---|---|---|---|---|---|---|---|---|---|---|---|---|---|
| | | | | **CLSI Cut-off Values (μg/mL)** | N/A | S≤4/8 R≥16/32 | S≤8 R≥32 | S≤8 R≥32 | S≤8 R≥64 | S≤2 R≥8 | S≤4 R≥16 | S≤2 R≥8 | S≤2 R≥8 | S≤4 R≥16 | S≤2/38 R≥4/76 |
| **IHMA Clinical Isolate #** | **Organism** | **Year Collected** | **Country** | **Body Location** | **OMN6** | **SAM (2:1)** | **FEP** | **CAZ** | **CRO** | **COL** | **GEN** | **LVX** | **MEM** | **TET** | **TSX (1:19)** |
| 2014367 | *A. baumannii* | 2019 | Jordan | Genitourinary: Urine | 4 | 2 | 1 | 4 | 8 | ≤0.12 | 0.25 | ≤0.12 | ≤0.06 | 0.25 | 0.12 |
| 2035493 | *A. baumannii* | 2019 | Ukraine | Cardiovascular: Blood | 4 | 64 | 64 | 16 | >64 | 0.25 | >16 | 4 | >16 | 4 | 1 |
| 1987378 | *A. baumannii* | 2019 | Georgia | Respiratory: Endotracheal aspirate | 4 | >64 | >64 | 32 | >64 | 0.25 | >16 | 16 | >16 | 2 | 4 |
| 1987388 | *A. baumannii* | 2019 | Georgia | Respiratory: Endotracheal aspirate | 8 | 64 | >64 | 32 | >64 | 0.25 | >16 | 32 | >16 | 2 | 2 |
| 2035462 | *A. baumannii* | 2019 | Ukraine | Respiratory: Endotracheal aspirate | 8 | 64 | >64 | 64 | >64 | 0.25 | 8 | 16 | >16 | 1 | 8 |
| 1987290 | *A. baumannii* | 2019 | Georgia | Respiratory: Endotracheal aspirate | 4 | 32 | >64 | 32 | >64 | 2 | >16 | 16 | >16 | 4 | 4 |
| 2028591 | *A. baumannii* | 2019 | United Kingdom | Cardiovascular: Blood | 8 | ≤1 | 1 | 2 | 4 | 0.5 | 0.5 | ≤0.12 | ≤0.06 | 0.25 | ≤0.06 |
| 1987331 | *A. baumannii* | 2019 | Georgia | Respiratory: Endotracheal aspirate | 4 | 64 | >64 | 32 | 64 | 0.25 | >16 | 8 | >16 | 0.5 | 1 |
| 1975524 | *A. baumannii* | 2019 | Israel | Respiratory: Sputum | 4 | 16 | 64 | >64 | >64 | 0.25 | 8 | 4 | 16 | >32 | >32 |
| 1987383 | *A. baumannii* | 2019 | Georgia | Respiratory: Endotracheal aspirate | 4 | >64 | >64 | 32 | >64 | 0.25 | >16 | 32 | >16 | 1 | 4 |
| 1975052 | *A. baumannii* | 2019 | Israel | Bodily Fluids: Thoracentesis | 8 | 32 | >64 | >64 | >64 | 0.25 | 16 | 8 | >16 | >32 | >32 |

| | | | | MIC values (µg/mL) | | | | | | | | | | | |
|---|---|---|---|---|---|---|---|---|---|---|---|---|---|---|---|
| | | | | **CLSI Cut-off Values (µg/mL)** | N/A | S≤4/8 R≥16/32 | S≤8 R≥32 | S≤8 R≥32 | S≤8 R≥64 | S≤2 R≥8 | S≤4 R≥16 | S≤2 R≥8 | S≤2 R≥8 | S≤4 R≥16 | S≤2/38 R≥4/76 |
| **IHMA Clinical Isolate #** | **Organism** | **Year Collected** | **Country** | **Body Location** | **OMN6** | **SAM (2:1)** | **FEP** | **CAZ** | **CRO** | **COL** | **GEN** | **LVX** | **MEM** | **TET** | **TSX (1:19)** |
| 1996909 | *A. baumannii* | 2019 | Kuwait | Respiratory: Endotracheal aspirate | **4** | 8 | 32 | >64 | >64 | 0.25 | 0.5 | 1 | 16 | >32 | 32 |
| 1975075 | *A. baumannii* | 2019 | Israel | Cardiovascular: Blood | **4** | 4 | 2 | 2 | 16 | 0.25 | 0.25 | ≤0.12 | 0.12 | 2 | 0.12 |
| 1977081 | *A. baumannii* | 2019 | Kuwait | Respiratory: Sputum | **4** | 16 | 32 | 64 | >64 | 0.25 | 0.5 | 1 | >16 | >32 | 32 |
| 1969002 | *A. baumannii* | 2019 | Israel | Respiratory: Bronchoalveolar lavage | **4** | 64 | 32 | >64 | >64 | 0.25 | >16 | 8 | >16 | 8 | 1 |
| 2129362 | *A. baumannii* | 2019 | Qatar | Respiratory: Sputum | **4** | 2 | 2 | 4 | 32 | 0.25 | 0.25 | 8 | 0.5 | >32 | 8 |
| 2020247 | *A. baumannii* | 2019 | Israel | Respiratory: Endotracheal aspirate | **4** | 4 | 4 | 4 | 16 | 0.25 | 0.5 | ≤0.12 | 0.5 | 1 | 0.25 |
| 2090519 | *A. baumannii* | 2019 | United States | Respiratory: Sputum | **8** | ≤1 | 1 | 2 | 8 | 0.25 | 0.25 | ≤0.12 | ≤0.06 | 0.5 | 0.25 |
| 2014436 | *A. baumannii* | 2019 | Jordan | Cardiovascular: Blood | **8** | >64 | >64 | >64 | >64 | 1 | 1 | 8 | >16 | 2 | 4 |
| 2000834 | *A. baumannii* | 2019 | United States | Respiratory: Bronchoalveolar lavage | **4** | 2 | 8 | 4 | 16 | 0.25 | 2 | ≤0.12 | 0.25 | 1 | ≤0.06 |
| 2014387 | *A. baumannii* | 2019 | Jordan | Respiratory: Sputum | **8** | 32 | 64 | >64 | >64 | 0.5 | >16 | 32 | >16 | 8 | 2 |
| 2088276 | *A. baumannii* | 2019 | United States | Respiratory: Sputum | **8** | 2 | 0.5 | 2 | 8 | 0.5 | 0.5 | ≤0.12 | 0.12 | >32 | 0.12 |
| 1975118 | *A. baumannii* | 2019 | Israel | Respiratory: Sputum | **4** | 2 | 2 | 4 | 16 | 0.25 | 0.25 | ≤0.12 | 0.25 | 1 | 0.12 |

| | | | | MIC values (µg/mL) | | | | | | | | | | | |
|---|---|---|---|---|---|---|---|---|---|---|---|---|---|---|---|
| | | | | CLSI Cut-off Values (µg/mL) | N/A | S≤4/8 R≥16/32 | S≤8 R≥32 | S≤8 R≥32 | S≤8 R≥64 | S≤2 R≥8 | S≤4 R≥16 | S≤2 R≥8 | S≤2 R≥8 | S≤4 R≥16 | S≤2/38 R≥4/76 |
| **IHMA Clinical Isolate #** | **Organism** | **Year Collected** | **Country** | **Body Location** | **OMN6** | **SAM (2:1)** | **FEP** | **CAZ** | **CRO** | **COL** | **GEN** | **LVX** | **MEM** | **TET** | **TSX (1:19)** |
| 1977086 | *A. baumannii* | 2019 | Kuwait | Respiratory: Endotracheal aspirate | 4 | 32 | 32 | >64 | >64 | 0.25 | >16 | 8 | >16 | >32 | 1 |
| 1964755 | *A. baumannii* | 2019 | United States | Bodily Fluids: Peritoneal | 4 | 2 | 1 | 4 | 16 | ≤0.12 | 0.25 | ≤0.12 | 0.12 | 1 | 0.12 |
| 1975603 | *A. baumannii* | 2019 | Israel | Respiratory: Sputum | 8 | 32 | 64 | >64 | >64 | 0.25 | >16 | 8 | >16 | >32 | 1 |
| 1996748 | *A. baumannii* | 2019 | Kuwait | Bodily Fluids: Abscess / Pus | 4 | 16 | 32 | 64 | >64 | 0.25 | 0.5 | 2 | >16 | >32 | 32 |
| 2088411 | *A. baumannii* | 2019 | United States | Respiratory: Endotracheal aspirate | 4 | 2 | 2 | 4 | 16 | ≤0.12 | 0.5 | ≤0.12 | 0.25 | 0.5 | 0.25 |
| 1975128 | *A. baumannii* | 2019 | Israel | Respiratory: Sputum | 4 | 16 | 32 | 64 | >64 | 0.25 | >16 | 2 | >16 | 2 | 32 |
| 2020244 | *A. baumannii* | 2019 | Israel | Respiratory: Endotracheal aspirate | 8 | 16 | 64 | 16 | 32 | 0.25 | ≤0.12 | 8 | 16 | 1 | 0.5 |
| 1996742 | *A. baumannii* | 2019 | Kuwait | Genitourinary: Urine | 4 | 8 | 16 | 32 | >64 | 0.25 | 0.5 | 2 | 8 | >32 | >32 |
| 1998890 | *A. baumannii* | 2019 | United States | Respiratory: Sputum | 4 | 8 | 4 | 32 | >64 | 0.25 | 1 | 16 | 8 | >32 | 0.25 |
| 1963202 | *A. baumannii* | 2019 | Israel | Respiratory: Sputum | 4 | 2 | 2 | 4 | 8 | 0.25 | 0.25 | ≤0.12 | 0.12 | 0.5 | 0.12 |
| 2046028 | *A. baumannii* | 2019 | Lebanon | Genitourinary: Urine | 8 | 64 | 64 | >64 | >64 | 0.5 | >16 | 8 | >16 | 8 | 1 |
| 2020257 | *A. baumannii* | 2019 | Israel | Respiratory: Endotracheal aspirate | 4 | 64 | >64 | >64 | >64 | 0.25 | >16 | 0.5 | 4 | 1 | >32 |

| | | | | MIC values (µg/mL) | | | | | | | | | | | |
|---|---|---|---|---|---|---|---|---|---|---|---|---|---|---|---|
| | | | | **CLSI Cut-off Values (µg/mL)** | N/A | S≤4/8 R≥16/32 | S≤8 R≥32 | S≤8 R≥32 | S≤8 R≥64 | S≤2 R≥8 | S≤4 R≥16 | S≤2 R≥8 | S≤2 R≥8 | S≤4 R≥16 | S≤2/38 R≥4/76 |
| **IHMA Clinical Isolate #** | **Organism** | **Year Collected** | **Country** | **Body Location** | **OMN6** | **SAM (2:1)** | **FEP** | **CAZ** | **CRO** | **COL** | **GEN** | **LVX** | **MEM** | **TET** | **TSX (1:19)** |
| 1992307 | *A. baumannii* | 2019 | United States | Respiratory: Endotracheal aspirate | 4 | 2 | 2 | 4 | 16 | ≤0.12 | 0.5 | ≤0.12 | 2 | 0.5 | ≤0.06 |
| 1963174 | *A. baumannii* | 2019 | Israel | Respiratory: Sputum | 4 | 4 | 8 | 64 | >64 | ≤0.12 | 0.5 | 4 | 0.5 | 4 | 8 |
| 2129302 | *A. baumannii* | 2019 | Qatar | Respiratory: Sputum | 4 | 16 | 32 | >64 | >64 | ≤0.12 | >16 | 4 | 16 | >32 | ≤0.06 |
| 1969045 | *A. baumannii* | 2019 | Israel | Respiratory: Sputum | 4 | 16 | 16 | 64 | >64 | 0.25 | >16 | 8 | >16 | >32 | >32 |
| 2082231 | *A. baumannii* | 2019 | United States | Respiratory: Endotracheal aspirate | 4 | ≤1 | 0.5 | 1 | 4 | 0.25 | 0.25 | ≤0.12 | ≤0.06 | 0.25 | 0.12 |
| 2082762 | *A. baumannii* | 2019 | United States | Respiratory: Lungs | 4 | 32 | 32 | 64 | >64 | ≤0.12 | >16 | 4 | >16 | >32 | 32 |
| 1957872 | *A. baumannii* | 2019 | Israel | Respiratory: Bronchials | 4 | 16 | 64 | >64 | >64 | 0.25 | 16 | 4 | 16 | >32 | >32 |
| 1975714 | *A. baumannii* | 2019 | Israel | Bodily Fluids: Peritoneal | 4 | 4 | 8 | >64 | >64 | ≤0.12 | 1 | 2 | 0.5 | 4 | 8 |
| 2082184 | *A. baumannii* | 2019 | United States | Respiratory: Endotracheal aspirate | 4 | ≤1 | 0.5 | 2 | 8 | 0.25 | 0.5 | ≤0.12 | 0.12 | 0.25 | 0.12 |
| 2020240 | *A. baumannii* | 2019 | Israel | Respiratory: Endotracheal aspirate | 4 | 2 | 2 | 8 | 16 | 0.25 | 0.5 | ≤0.12 | 0.12 | 0.5 | ≤0.06 |
| 2090615 | *A. baumannii* | 2019 | United States | Respiratory: Sputum | 4 | 16 | 32 | 32 | >64 | 0.25 | 0.5 | 8 | 16 | >32 | 32 |
| 2020248 | *A. baumannii* | 2019 | Israel | Respiratory: Sputum | 8 | 4 | 8 | 16 | >64 | 0.25 | >16 | 0.5 | 1 | >32 | 4 |

| | | | | MIC values (µg/mL) | | | | | | | | | | | |
|---|---|---|---|---|---|---|---|---|---|---|---|---|---|---|---|
| | | | | **CLSI Cut-off Values (µg/mL)** | N/A | S≤4/8 R≥16/32 | S≤8 R≥32 | S≤8 R≥32 | S≤8 R≥64 | S≤2 R≥8 | S≤4 R≥16 | S≤2 R≥8 | S≤2 R≥8 | S≤4 R≥16 | S≤2/38 R≥4/76 |
| **IHMA Clinical Isolate #** | **Organism** | **Year Collected** | **Country** | **Body Location** | **OMN6** | **SAM (2:1)** | **FEP** | **CAZ** | **CRO** | **COL** | **GEN** | **LVX** | **MEM** | **TET** | **TSX (1:19)** |
| 2026189 | *A. baumannii* | 2019 | Australia | Genitourinary: Urine | 4 | ≤1 | 0.5 | 1 | 8 | ≤0.12 | 1 | ≤0.12 | ≤0.06 | 0.25 | 0.12 |
| 2014437 | *A. baumannii* | 2019 | Jordan | Respiratory: Sputum | 8 | 32 | >64 | 64 | >64 | 0.5 | >16 | 16 | >16 | 8 | >32 |
| 2019036 | *A. baumannii* | 2019 | United States | Respiratory: Sputum | 8 | 16 | 32 | 64 | >64 | 0.25 | >16 | 2 | >16 | >32 | 32 |
| 1968943 | *A. baumannii* | 2019 | Israel | Cardiovascular: Blood | 4 | 64 | >64 | >64 | >64 | 0.25 | 2 | 4 | >16 | >32 | 0.5 |
| 1990668 | *A. baumannii* | 2019 | Philippines | Bodily Fluids: Peritoneal | 4 | 64 | >64 | >64 | >64 | 0.25 | >16 | 32 | >16 | 4 | 32 |
| 1977074 | *A. baumannii* | 2019 | Kuwait | Genitourinary: Urine | 4 | 8 | 64 | >64 | >64 | 0.25 | >16 | 2 | 0.25 | >32 | ≤0.06 |
| 2020254 | *A. baumannii* | 2019 | Israel | Respiratory: Endotracheal aspirate | 4 | 2 | 2 | 4 | 16 | ≤0.12 | 0.5 | ≤0.12 | 0.25 | 1 | 0.25 |
| 2088269 | *A. baumannii* | 2019 | United States | Respiratory: Bronchoalveolar lavage | 8 | 2 | 2 | 4 | 16 | 0.25 | 1 | ≤0.12 | 0.12 | 0.5 | 0.25 |
| 2004158 | *A. baumannii* | 2019 | Philippines | Respiratory: Endotracheal aspirate | 4 | 64 | >64 | >64 | >64 | 0.25 | >16 | 16 | >16 | 4 | 32 |
| 1996891 | *A. baumannii* | 2019 | Kuwait | Respiratory: Endotracheal aspirate | 4 | 64 | 64 | >64 | >64 | 0.5 | >16 | 2 | >16 | >32 | 16 |
| 2020255 | *A. baumannii* | 2019 | Israel | Respiratory: Endotracheal aspirate | 4 | 2 | 1 | 2 | 16 | 0.25 | 0.5 | ≤0.12 | 0.12 | 0.25 | ≤0.06 |
| 2053338 | *A. baumannii* | 2019 | United States | Respiratory: Sputum | 8 | 32 | 16 | >64 | >64 | 0.25 | >16 | 4 | >16 | >32 | 8 |

| | | | | MIC values (µg/mL) | | | | | | | | | | | |
|---|---|---|---|---|---|---|---|---|---|---|---|---|---|---|---|
| | | | | **CLSI Cut-off Values (µg/mL)** | N/A | S≤4/8 R≥16/32 | S≤8 R≥32 | S≤8 R≥32 | S≤8 R≥64 | S≤2 R≥8 | S≤4 R≥16 | S≤2 R≥8 | S≤2 R≥8 | S≤4 R≥16 | S≤2/38 R≥4/76 |
| **IHMA Clinical Isolate #** | **Organism** | **Year Collected** | **Country** | **Body Location** | **OMN6** | **SAM (2:1)** | **FEP** | **CAZ** | **CRO** | **COL** | **GEN** | **LVX** | **MEM** | **TET** | **TSX (1:19)** |
| 1984887 | *A. baumannii* | 2019 | Philippines | Respiratory: Sputum | 4 | 64 | 64 | >64 | >64 | 0.25 | 1 | 16 | >16 | 4 | >32 |
| 2014441 | *A. baumannii* | 2019 | Jordan | Respiratory: Sputum | 4 | 8 | 8 | 16 | >64 | 4 | >16 | >32 | 16 | >32 | 32 |
| 1996925 | *A. baumannii* | 2019 | Kuwait | Respiratory: Endotracheal aspirate | 4 | 16 | 32 | 32 | >64 | 0.25 | 0.25 | 2 | >16 | >32 | 32 |
| 1963468 | *A. baumannii* | 2019 | United States | Bodily Fluids: Peritoneal | 4 | 2 | 1 | 4 | 16 | ≤0.12 | ≤0.12 | ≤0.12 | 0.12 | 0.5 | 0.12 |
| 1976951 | *A. baumannii* | 2019 | Kuwait | Bodily Fluids: Abscess / Pus | 4 | 8 | 16 | 32 | >64 | 0.25 | 0.5 | 2 | 16 | >32 | 32 |
| 1996809 | *A. baumannii* | 2019 | Kuwait | Cardiovascular: Blood | 4 | 64 | 64 | >64 | >64 | 1 | >16 | 2 | >16 | >32 | 16 |
| 2082626 | *A. baumannii* | 2019 | United States | Respiratory: Sputum | 8 | 8 | 16 | >64 | >64 | 0.25 | >16 | 8 | >16 | 4 | 8 |
| 1977085 | *A. baumannii* | 2019 | Kuwait | Gastrointestinal: Rectum | 4 | 8 | 16 | 64 | >64 | 0.25 | 0.5 | 2 | 16 | >32 | 16 |
| 1996863 | *A. baumannii* | 2019 | Kuwait | Respiratory: Endotracheal aspirate | 4 | 32 | 64 | 64 | >64 | 0.25 | 0.25 | 2 | >16 | >32 | 16 |
| 1992310 | *A. baumannii* | 2019 | United States | Genitourinary: Urine | 4 | ≤1 | 16 | 8 | 16 | ≤0.12 | 0.25 | 0.25 | 0.12 | 0.5 | ≤0.06 |
| 2129359 | *A. baumannii* | 2019 | Qatar | Respiratory: Sputum | 4 | ≤1 | 0.5 | 1 | 4 | 0.25 | 0.25 | ≤0.12 | ≤0.06 | 0.25 | 0.12 |
| 2036956 | *A. baumannii* | 2019 | Lebanon | Genitourinary: Urine | 4 | 32 | 64 | >64 | >64 | 0.25 | >16 | 8 | >16 | >32 | 32 |
| 2088470 | *A. baumannii* | 2019 | United States | Respiratory: Sputum | 8 | 16 | 32 | >64 | >64 | 0.25 | 2 | 8 | 16 | 1 | 0.12 |

| | | | | | MIC values (µg/mL) | | | | | | | | | | |
|---|---|---|---|---|---|---|---|---|---|---|---|---|---|---|---|
| | | | | CLSI Cut-off Values (µg/mL) | N/A | S≤4/8 R≥16/32 | S≤8 R≥32 | S≤8 R≥32 | S≤8 R≥64 | S≤2 R≥8 | S≤4 R≥16 | S≤2 R≥8 | S≤2 R≥8 | S≤4 R≥16 | S≤2/38 R≥4/76 |
| **IHMA Clinical Isolate #** | **Organism** | **Year Collected** | **Country** | **Body Location** | **OMN6** | **SAM (2:1)** | **FEP** | **CAZ** | **CRO** | **COL** | **GEN** | **LVX** | **MEM** | **TET** | **TSX (1:19)** |
| 2129256 | *A. baumannii* | 2019 | Qatar | Bodily Fluids: Peritoneal | 4 | ≤1 | 1 | 2 | 8 | 0.25 | 0.25 | ≤0.12 | ≤0.06 | 0.25 | ≤0.06 |
| 2090509 | *A. baumannii* | 2019 | United States | Respiratory: Bronchoalveolar lavage | 8 | >64 | 32 | 32 | >64 | 0.25 | >16 | 16 | >16 | >32 | >32 |
| 2084306 | *A. baumannii* | 2019 | United States | Respiratory: Sputum | 8 | 8 | 16 | 32 | >64 | 0.25 | >16 | 4 | 4 | >32 | >32 |
| 2115382 | *A. baumannii* | 2019 | United States | Genitourinary: Urine | 4 | 2 | 2 | 4 | 16 | 0.25 | 0.5 | ≤0.12 | 0.25 | 0.5 | 0.12 |
| 2041470 | *A. baumannii* | 2019 | United States | Respiratory: Endotracheal aspirate | 4 | 32 | 64 | >64 | >64 | 0.25 | >16 | 8 | >16 | >32 | 32 |
| 2020680 | *A. baumannii* | 2019 | Australia | Genitourinary: Urine | 4 | ≤1 | 1 | 1 | 16 | 0.25 | 1 | ≤0.12 | 0.12 | 0.25 | ≤0.06 |
| 2024881 | *A. baumannii* | 2019 | United States | Respiratory: Other | 4 | 32 | 16 | 64 | >64 | 0.25 | >16 | 4 | >16 | >32 | 32 |
| 2092806 | *A. baumannii* | 2019 | United States | Genitourinary: Urine | 4 | 2 | 1 | 4 | 16 | 0.25 | 0.5 | ≤0.12 | ≤0.06 | 0.25 | 0.12 |
| 1984493 | *A. baumannii* | 2019 | Australia | Respiratory: Sputum | 4 | 2 | 1 | 4 | 16 | 0.25 | 0.5 | ≤0.12 | 0.12 | 0.25 | 0.25 |
| 2090612 | *A. baumannii* | 2019 | United States | Respiratory: Bronchoalveolar lavage | 4 | 64 | 32 | 32 | >64 | 0.25 | >16 | 16 | >16 | >32 | >32 |
| 2019219 | *A. baumannii* | 2019 | United States | Respiratory: Endotracheal aspirate | 4 | 8 | 16 | >64 | >64 | 0.25 | 4 | 32 | >16 | 4 | 8 |
| 1986328 | *A. baumannii* | 2019 | United States | Genitourinary: Urine | 4 | 2 | 1 | 2 | 8 | 0.25 | 0.5 | ≤0.12 | ≤0.06 | 0.25 | 0.12 |

| IHMA Clinical Isolate # | Organism | Year Collected | Country | Body Location | OMN6 | SAM (2:1) | FEP | CAZ | CRO | COL | GEN | LVX | MEM | TET | TSX (1:19) |
|---|---|---|---|---|---|---|---|---|---|---|---|---|---|---|---|
| | | | | | | **MIC values (µg/mL)** | | | | | | | | | |
| | | | | **CLSI Cut-off Values (µg/mL)** | N/A | S≤4/8 R≥16/32 | S≤8 R≥32 | S≤8 R≥32 | S≤8 R≥64 | S≤2 R≥8 | S≤4 R≥16 | S≤2 R≥8 | S≤2 R≥8 | S≤4 R≥16 | S≤2/38 R≥4/76 |
| 2092731 | *A. baumannii* | 2019 | United States | Respiratory: Other | 4 | 16 | 32 | >64 | >64 | 0.25 | >16 | 16 | >16 | 4 | >32 |
| 2004640 | *A. baumannii* | 2019 | United States | Respiratory: Sputum | 4 | 2 | 1 | 2 | 8 | ≤0.12 | 0.25 | ≤0.12 | ≤0.06 | 0.25 | ≤0.06 |
| 2000832 | *A. baumannii* | 2019 | United States | Respiratory: Bronchoalveolar lavage | 4 | 2 | 2 | 2 | 16 | 0.25 | 0.25 | ≤0.12 | 0.12 | 0.5 | 0.25 |
| 2043615 | *A. baumannii* | 2019 | United States | Genitourinary: Urine | 4 | 4 | 32 | >64 | >64 | 0.25 | >16 | 16 | >16 | 4 | >32 |
| 2088270 | *A. baumannii* | 2019 | United States | Respiratory: Bronchoalveolar lavage | 4 | ≤1 | 8 | 4 | 8 | 0.25 | >16 | ≤0.12 | 0.25 | 0.5 | 0.12 |
| 2092807 | *A. baumannii* | 2019 | United States | Respiratory: Endotracheal aspirate | 4 | 32 | 32 | >64 | >64 | 0.25 | 0.5 | 16 | >16 | 2 | 0.5 |
| 2082625 | *A. baumannii* | 2019 | United States | Respiratory: Endotracheal aspirate | 8 | 64 | 8 | 32 | >64 | 0.25 | >16 | 4 | 1 | >32 | >32 |
| 2111440 | *A. baumannii* | 2019 | United States | Genitourinary: Urine | 4 | 16 | 16 | 64 | >64 | ≤0.12 | >16 | 2 | >16 | >32 | >32 |
| 2084326 | *A. baumannii* | 2019 | United States | Respiratory: Sputum | 4 | 8 | 16 | 32 | >64 | 0.25 | >16 | 4 | 4 | >32 | >32 |
| 2053330 | *A. baumannii* | 2019 | United States | Respiratory: Bronchoalveolar lavage | 8 | 32 | 32 | >64 | >64 | 0.25 | >16 | 2 | >16 | >32 | 0.25 |
| 2084337 | *A. baumannii* | 2019 | United States | Respiratory: Endotracheal aspirate | 8 | 8 | 16 | >64 | >64 | 1 | 0.25 | 32 | 16 | 8 | 0.5 |

| | | | | MIC values (µg/mL) | | | | | | | | | | | |
|---|---|---|---|---|---|---|---|---|---|---|---|---|---|---|---|
| | | | | **CLSI Cut-off Values (µg/mL)** | N/A | S≤4/8 R≥16/32 | S≤8 R≥32 | S≤8 R≥32 | S≤8 R≥64 | S≤2 R≥8 | S≤4 R≥16 | S≤2 R≥8 | S≤2 R≥8 | S≤4 R≥16 | S≤2/38 R≥4/76 |
| **IHMA Clinical Isolate #** | **Organism** | **Year Collected** | **Country** | **Body Location** | **OMN6** | **SAM (2:1)** | **FEP** | **CAZ** | **CRO** | **COL** | **GEN** | **LVX** | **MEM** | **TET** | **TSX (1:19)** |
| 2082025 | *A. baumannii* | 2019 | United States | Genitourinary: Urine | **4** | 2 | >64 | >64 | 64 | ≤0.12 | 0.25 | 2 | 0.5 | >32 | 0.5 |
| 1988902 | *A. baumannii* | 2019 | United States | Respiratory: Sputum | **4** | 4 | >64 | >64 | >64 | 0.25 | >16 | 16 | >16 | 8 | >32 |
| 2088463 | *A. baumannii* | 2019 | United States | Respiratory: Sputum | **4** | ≤1 | 1 | 1 | 4 | 0.25 | 0.5 | ≤0.12 | ≤0.06 | 0.25 | 0.12 |
| 2020647 | *A. baumannii* | 2019 | Australia | Bodily Fluids: Peritoneal | **4** | 2 | 1 | 2 | 8 | ≤0.12 | 0.25 | ≤0.12 | 0.12 | 0.5 | ≤0.06 |
| 1988057 | *A. baumannii* | 2019 | United States | Respiratory: Sputum | **4** | 8 | 16 | 32 | >64 | 0.25 | >16 | 4 | 8 | >32 | >32 |
| 1984885 | *A. baumannii* | 2019 | Philippines | Respiratory: Endotracheal aspirate | **8** | 32 | 64 | >64 | >64 | 0.25 | >16 | 16 | >16 | 4 | 32 |
| 2115543 | *A. baumannii* | 2019 | United States | Genitourinary: Urine | **4** | ≤1 | 1 | 2 | 8 | ≤0.12 | 0.5 | ≤0.12 | ≤0.06 | 0.5 | 0.12 |
| 1984888 | *A. baumannii* | 2019 | Philippines | Respiratory: Endotracheal aspirate | **4** | 4 | 4 | 4 | 32 | 0.25 | 0.5 | ≤0.12 | 0.25 | 1 | 0.12 |
| 1988946 | *A. baumannii* | 2019 | United States | Respiratory: Sputum | **8** | 16 | 16 | 64 | >64 | 0.25 | >16 | 8 | >16 | >32 | >32 |
| 2104368 | *A. baumannii* | 2019 | Australia | Genitourinary: Urine | **4** | ≤1 | ≤0.25 | ≤0.25 | 1 | ≤0.12 | 0.25 | ≤0.12 | ≤0.06 | ≤0.12 | ≤0.06 |
| 2142427 | *A. baumannii* | 2019 | Australia | Bodily Fluids: Peritoneal | **4** | ≤1 | ≤0.25 | 0.5 | 4 | ≤0.12 | 0.5 | ≤0.12 | ≤0.06 | 0.5 | 0.12 |
| 2104351 | *A. baumannii* | 2019 | Australia | Genitourinary: Urine | **4** | 2 | 2 | 4 | 16 | 0.25 | 0.5 | ≤0.12 | 0.12 | 0.5 | 0.12 |

**Table S2. MIC distribution against 401 clinical isolates tested for OMN6 and other antibiotics.** Bacteria were subjected to MIC tests with the broth microdilution method by IHMA Europe Sàrl, following the principles outlined in the CLSI guidelines.

| | Clinical isolates numbers per MIC value (µg/mL) | | | | | | | | | | | | | | | | | |
|---|---|---|---|---|---|---|---|---|---|---|---|---|---|---|---|---|---|---|
| **Drug** | **≤0.06** | **≤0.12** | **0.12** | **≤0.25** | **0.25** | **0.5** | **≤1** | **1** | **2** | **4** | **8** | **>8** | **16** | **>16** | **32** | **>32** | **64** | **>64** |
| **OMN6** | | | | | | | | | | 288 | 113 | | | | | | | |
| **SAM (2:1)** | | | | | | | 43 | | 49 | 25 | 40 | | 63 | | 85 | | 71 | 25 |
| **FEP** | | | | 6 | | 15 | | 42 | 27 | 15 | 27 | | 50 | | 84 | | 82 | 53 |
| **CAZ** | | | | 1 | | 2 | | 20 | 40 | 43 | 5 | | 16 | | 31 | | 50 | 193 |
| **CRO** | | | | | | | | 2 | 7 | 11 | 41 | | 42 | | 7 | | 3 | 288 |
| **COL** | | 53 | | | 287 | 28 | | 15 | 7 | 2 | 1 | 8 | | | | | | |
| **GEN** | | 5 | | | 50 | 75 | | 18 | 14 | 6 | 5 | | 10 | 218 | | | | |
| **LVX** | | 101 | | | 1 | 3 | | 9 | 64 | 64 | 82 | | 39 | | 23 | 15 | | |
| **MEM** | 36 | | 44 | | 18 | 13 | | 5 | 4 | 6 | 16 | | 58 | 201 | | | | |
| **TET** | | 3 | | | 37 | 57 | | 34 | 26 | 32 | 13 | | 2 | | 3 | 194 | | |
| **SXT (1:19)** | 36 | | 67 | | 18 | 19 | | 33 | 15 | 20 | 24 | | 17 | | 61 | 91 | | |

**Table S3. Supplementary information about the *A. baumannii-calcoaceticus* species complex strains and clinical isolates tested in the serial passaging study presented in Figure 1 and the MIC study presented in Table 2.** The resistance phenotypes of the clinical isolates were characterized by JMI Laboratories (North Liberty, IA, USA) using the broth microdilution method following the principles outlined in the CLSI guidelines. The resistance phenotypes were determined according to CLSI interpretive criteria for meropenem and EUCAST criteria for colistin. Abbreviations: Col-S, colistin-susceptible; Col-R, colistin-resistant; Mer-S, meropenem-susceptible; Mer-R, meropenem-resistant; ATCC, American Type Culture Collection; BPS, Bovine Pulmonary Surfactant; NCTC, National Collection of Type Cultures; N/A, not applicable.

| Collection No. | Relevant phenotypes [a] | Continent | Serial passaging study | BPS MIC study |
|---|---|---|---|---|
| **ATCC 19606** | Col-S Mer-S | N/A | Yes | |
| **NCTC 13304** | Col-S Mer-R | N/A | Yes | |
| **1092865** [b] | Col-S Mer-S | Europe | | Yes |
| **1094885** [b] | Col-S Mer-S | North America | Yes | Yes |
| **1095725** [b] | Col-S Mer-R | North America | | Yes |
| **1096848** [b] | Col-S Mer-R | Europe | Yes | Yes |
| **1098733** [b] | Col-S Mer-S | Europe | | Yes |
| **1111408** [b] | Col-R Mer-S | North America | | Yes |
| **1111669** [b] | Col-R Mer-R | North America | | Yes |
| **1115893** [b] | Col-R Mer-S | North America | | Yes |
| **1115918** [b] | Col-S Mer-S | North America | | Yes |
| **1128135** [b] | Col-R Mer-R | Europe | | Yes |

[a] EUCAST breakpoint interpretive criteria were used.

[b] Clinical isolate obtained from the 2019 JMI Laboratories SENTRY program.

**Table S4. Supplementary information about the resistance pattern of the bacterial strain *A. baumannii* BAA-1793 used in the mouse bacteremia survival model presented in Figure 2.** The antibiogram was determined by ATCC according to CLSI guidelines, and is presented in the next table according to the following abbreviations: *S* Susceptible, *I* intermediate, *R* resistant.

| Antibiotic name | Resistance pattern | Antibiotic name | Resistance pattern |
|---|---|---|---|
| Amikacin | S | Ciprofloxacin | R |
| Amoxicillin / Clavulanic acid | R | Gentamicin | S |
| Ampicillin | R | Imipenem | R |
| Ampicillin/Sulbactam | S | Levofloxacin | I |
| Aztreonam | R | Meropenem | R |
| Cefalotin | R | Moxifloxacin | R |
| Cefazolin | R | Nalidixic Acid | R |
| Cefepime | R | Nitrofurantoin | R |
| Cefotaxime | R | Norfloxacin | R |
| Cefotetan | R | Piperacillin | R |
| Cefoxitin | R | Piperacillin / Tazobactam | R |
| Cefpodoxime | R | Tetracycline | R |
| Ceftazidime | R | Ticarcillin | R |
| Ceftizoxime | R | Ticarcillin / Clavulanic acid | R |
| Ceftriaxone | R | Tigecycline | S |
| Cefuroxime | R | Tobramycin | R |
| Cefuroxime Axetil | R | Trimethoprim / Sulfamethoxazole | S |

**Table S5. Supplementary information about the resistance pattern of the *A. baumannii* ACC000535 clinical isolate used in the chronic lung infection model presented in Figure 3 and in the time-course study presented in Figure 4.** The resistance pattern of the different bacterial isolates was characterized by broth microdilution studies for colistin, or with the VITEK 2 (BioMérieux SA, Marcy l'Etoile, France) system for the other antibiotics. The resistance range of each antibiotic has been determined according to CLSI guidelines, and is presented in the next table according to the following abbreviations: *S* Susceptible, *I* intermediate, *R* resistant, *MDR* multidrug resistant, *MIC* Minimal Inhibitory Concentration, *GEN* gentamycin, *AUG* amoxicillin/clavulanic acid, *IMP* imipenem, *CTX* cefotaxime, *CIP* ciprofloxacin, *PTZ* piperacillin/tazobactam, *CAZ* ceftazidime.

| | | | | VITEK characterization | | | | | | |
|---|---|---|---|---|---|---|---|---|---|---|
| **Bacteria** | **Strain** | **Resistance pattern** | **Colistin MIC (µg/mL)** | **GEN** | **AUG** | **IMP** | **CTX** | **CIP** | **PTZ** | **CAZ** |
| *A. baumannii* | ACC 00535 | MDR | 0.25 | R | R | I | R | R | R | R |
